# Covid‐19, social restrictions, and mental distress among young people: a UK longitudinal, population‐based study

**DOI:** 10.1111/jcpp.13586

**Published:** 2022-02-23

**Authors:** Gemma Knowles, Charlotte Gayer‐Anderson, Alice Turner, Lynsey Dorn, Joseph Lam, Samantha Davis, Rachel Blakey, Katie Lowis, Schools Working Group, Young Persons Advisory Group, Vanessa Pinfold, Natalie Creary, Jacqui Dyer, Stephani L. Hatch, George Ploubidis, Kamaldeep Bhui, Seeromanie Harding, Craig Morgan

**Affiliations:** ^1^ Health Service and Population Research Department Institute of Psychiatry, Psychology and Neuroscience King’s College London London UK; ^2^ ESRC Centre for Society and Mental Health King’s College London London UK; ^3^ The McPin Foundation London UK; ^4^ Black Thrive London UK; ^5^ Psychological Medicine Institute of Psychiatry, Psychology and Neuroscience King’s College London London UK; ^6^ Centre for Longitudinal Studies University College London London UK; ^7^ Department of Psychiatry University of Oxford Oxford UK; ^8^ Department of Nutritional Sciences School of Life Course Sciences Faculty of Life Sciences and Medicine King’s College London London UK

**Keywords:** Covid‐19, mental distress, adolescence, cohort

## Abstract

**Background:**

Adolescence is a critical period for social and emotional development. We sought to examine the impacts of Covid‐19 and related social restrictions and school closures on adolescent mental health, particularly among disadvantaged, marginalised, and vulnerable groups.

**Methods:**

We analysed four waves of data – 3 pre‐Covid‐19 (2016–2019) and 1 mid‐Covid‐19 (May–Aug 2020; *n*, 1074; 12–18 years old, >80% minority ethnic groups, 25% free school meals) from REACH (Resilience, Ethnicity, and AdolesCent Mental Health), an adolescent cohort based in inner‐London, United Kingdom. Mental health was assessed using validated measures at each time point. We estimated temporal trends in mental distress and examined variations in changes in distress, pre‐ to mid‐Covid‐19, by social group, and by pre‐ and mid‐pandemic risks.

**Results:**

We found no evidence of an overall increase in mental distress midpandemic (15.9%, 95% CI: 13.0, 19.4) compared with prepandemic (around 18%). However, there were variations in changes in mental distress by subgroups. There were modest variations by social group and by pre‐Covid risks (e.g., a small increase in distress among girls (b [unstandardised beta coefficient] 0.42 [−0.19, 1.03]); a small decrease among boys (b − 0.59 [−1.37, 0.19]); *p* for interaction .007). The most notable variations were by midpandemic risks: that is, broadly, increases in distress among those reporting negative circumstances and impacts (e.g., in finances, housing, social support and relationships, and daily routines) and decreases in distress among those reporting positive impacts.

**Conclusions:**

We found strong evidence that mental distress increased among young people who were most negatively impacted by Covid‐19 and by related social restrictions during the first lockdown in the United Kingdom.

## Introduction

Adolescence is a critical period of social and emotional development (Rapee et al., 2019), during which normal developmental processes, such as hormonal changes, difficulties with emotion regulation, and salience of peer relationships, mean young people are especially susceptible to socioemotional problems (e.g., anxiety and depression) when faced with challenging contexts. Further, there is evidence that rates of mental distress have increased among adolescents in the past 20 years, particularly among girls (Keyes, Gary, O’Malley, Hamilton, & Schulenberg, 2019; Patalay & Gage, 2019). Explanations for this have centred on increased pressures related to exams, the rise of social media, and the impacts of rising inequality and poverty (Bor, Dean, Najman, & Hayatbakhsh, 2014; Collishaw, 2015).

It is against this background that there is concern about the impacts of Covid‐19 on adolescent mental health, especially for the most disadvantaged, marginalised, and vulnerable groups (Cowie & Myers, 2020; Major, Eyles, & Machin, 2020). School closures and social restrictions directly impact aspects of young people’s lives already implicated in the occurrence of mental distress (e.g., exam pressures, peer relationships, and household poverty), amplifying these pressures and risks at a critical developmental stage, with potential lifelong consequences. There is particular concern about the impacts on mental health among those living in households directly affected (e.g., by parental job loss; by financial, food, housing insecurity, and so on), among those vulnerable to abuse and violence, among girls, and among young people from minority ethnic backgrounds. For example, minority ethnic (racial) communities have been impacted more by issues related to housing, finances, and employment during the pandemic (Mind, 2020), and have experienced higher rates of infection and death from Covid‐19 (ONS, 2021).

Some relevant evidence has been published. Many surveys have been conducted, some with repeated measures at several (midpandemic) time points. However, findings are inconsistent across studies. Some surveys suggest the mental health of young people worsened, particularly among specific subgroups (e.g., those with preexisting mental health problems) (Young Minds, 2020), and others suggest mental health has remained constant or even improved slightly (Shum, Skripkauskaite, Pearcey, Waite, & Creswell, 2021). However, most early surveys were cross‐sectional, used convenience online samples, which are subject to selection biases, and did not have pre‐Covid‐19 data for comparison. More recent studies including pre‐ and post‐Covid‐19 comparisons have emerged (Magson et al., 2021; Newlove‐Delgado et al., 2021; Patalay & Fitzsimons, 2020; Vizard et al., 2020; Widnall, Winstone, Mars, Haworth, & Kidger, 2020), but findings are again mixed, methods varied, and samples relatively homogenous, with minority ethnic groups and those from low‐income households – that is, those most likely to be impacted by social restrictions and school closures – underrepresented. Further, a primary focus on overall trends neglects variations by social and ethnic group and by direct impacts of the pandemic, be these negative or positive.

Our study of adolescent mental health in inner‐London, the **R**esilience, **E**thnicity, and **A**doles**C**ent Mental **H**ealth (REACH) study, comprises socially and ethnically diverse cohorts, initially aged 11–14 years, followed annually for 3 years immediately prepandemic. To examine the impacts of the first period of social and economic restrictions in the United Kingdom on the mental health of young people from diverse backgrounds, we followed these cohorts midpandemic (i.e., during the first period of lockdown and school closures in the United Kingdom, May–August 2020).

We sought to examine variations in impacts on mental health by social and ethnic group, by preexisting risks (e.g., prior mental health problems), and by the direct consequences of Covid‐19, social restrictions, and school closures. We sought to test four primary hypotheses:

1. Mental distress increased between Time 1–3 (prepandemic) and Time 4 (midpandemic)
2. Increases in mental distress between T1–3 and T4 were greater among those in putative risk groups: (a) those with prior mental health problems; (b) girls; (c) those in low‐income households; and (d) minority ethnic groups
3. Increases in mental distress between T1–3 and T4 were greater among those (a) in putative risk groups and (b) who reported worse midpandemic circumstances
4. Changes in mental distress between T1‐3 and T4 varied by perceived impacts of the pandemic, with increases among those who reported negatives and decreases among those who reported positives

## Methods

REACH has been codesigned and implemented in partnership with young people and teachers. For T4, in March–April 2020, we conducted several focus groups and interviews with our Young Persons Advisory Group (YPAG) and Teacher Advisory Group (TAG) to shape our research questions, methods of recontact, and the content and wording of the questionnaire.

### Design, participants, and T4 procedures

REACH is an ongoing accelerated cohort study of adolescent mental health in two socially and ethnically diverse inner‐city London boroughs, Southwark and Lambeth, United Kingdom. The cohorts, local context, and prepandemic study procedures are described in detail elsewhere (Knowles et al., 2021). The REACH cohorts (total *n* 4,353, age 11–14 at inception) were recruited from twelve local secondary schools and are representative of secondary school pupils in the two boroughs (e.g., >80% minority ethnic groups). At T3, participants were provided with a ‘Consent to Contact’ form, providing options to be contacted about participation in future waves of data collection. For T4, we sought to recontact, reconsent, and collect data from all young people who had taken part in at least one prepandemic wave of REACH and who, by then, had provided recontact information (*n* 2,692).

### Data collection

Prepandemic, participants completed three waves of data collection at yearly intervals (T1–T3, 2016–2019). All completed a questionnaire that comprised validated schedules designed to elicit detailed information on demographic characteristics and social circumstances, mental health, and putative risk and protective factors (e.g., financial hardship, bullying victimisation, and peer and adult confidantes).

At T4, we revised the questionnaire, retaining core validated and widely used schedules on mental health and social circumstances. Overall and specific types of mental distress were assessed using the Strengths and Difficulties Questionnaire (SDQ) (Goodman, Meltzer, & Bailey, 1998), the Short Mood and Feelings Questionnaire (SFMQ) (Ancold & Stephen, 1995), the Generalised Anxiety Disorder Scale (GAD‐7) (Spitzer, Kroenke, Williams, & Löwe, 2006), and a single item on lifetime self‐harm (Goodman, Ford, Richards, Gatward, & Meltzer, 2000). We also added items related to the impacts and experiences of Covid‐19 by reviewing all emerging Covid‐19‐related mental health research to maximise comparability. We included items on (a) Covid‐19 infection; (b) housing space and quality; (c) household economic impacts; (d) relationships and supports; (e) lifestyle and daily routines; and (f) worries, concerns, and perceived positives. For full details, see Appendix S1.

#### Primary outcome measure

The self‐report SDQ is one of the most widely used and well‐validated measures for screening 11–17‐year‐olds for emotional and behavioural difficulties during the preceding 6 months (Goodman et al., 1998). Following established procedures (Goodman, Lamping, & Ploubidis, 2010), we calculated a total difficulties score (0–40) and internalising and externalising scores (0–20). Where relevant, total difficulties scores were categorised using established thresholds (≥18 indicating probable mental health problem) (Goodman, 2001).

Prior to completing the T4 questionnaire online, all young people provided electronic informed consent after reading information sheets. T4 data collection is ongoing. The analyses presented in this paper were conducted on those who participated between May and August 2020 (*n* = 1074), that is, before the reopening of schools in England.

All procedures were approved by the Psychiatry, Nursing and Midwifery Research Ethics Subcommittee (PNM‐RESC), King’s College London (ref:15/162320).

### Analyses

Analyses were conducted in three steps, following the approach used by Pierce and colleagues in their analyses of the acute impacts of the Covid‐19 pandemic on adult mental health (Pierce et al., 2020). First, we produced descriptive statistics for the T4 sample (*n*, 1074) and, to assess potential biases, compared the T4 sample with the full REACH cohort and the target population. To account for potential nonresponse bias we calculated inverse probability weights, as follows: (a) we selected putative predictors of nonresponse a priori and by comparing those who completed the T4 questionnaire with those who did not on core variables; (b) we modelled selected predictors (i.e., school year, gender, ethnicity, free school meals, high SDQ score at T1, T2, and T3, and interaction terms for gender and high SDQ scores) using multilevel logistic regression, checking model fit using Hosmer–Lemeshow goodness of fit test (*p* for all tests were >.1); (c) we used predicted probabilities to calculate weights (i.e., weight = 1/pr); and (d) after checking the range and distribution of weights, we truncated weights at 10 to address potential issues with large weights. We applied inverse probability weights to all subsequent models.

Second, to test Hypothesis 1, we estimated the (weighted) prevalence of mental health problems (overall and by type) and lifetime self‐harm at each wave (i.e., T1, T2, T3, and T4) in the sample that took part at T4 (sample size at T1: *n* 955; T2: *n* 943; and T3 *n* 958). Mental health problems were defined as SDQ scores ≥18, depression as SMFQ score ≥12, and moderate‐to‐severe anxiety as GAD‐7 score ≥10. We also calculated mean SDQ total difficulties, internalising, and externalising scores at each wave. 95% confidence intervals (CIs) were estimated using robust standard errors to account for the clustering of pupils within schools.

Third, to test Hypotheses 2–4, we used fixed effects (i.e., within‐person and time‐demeaned) regression (xtreg, fe command in Stata) to estimate the pre to midpandemic within‐person change in SDQ scores (i.e., change between T1–T3 and T4), overall and by (a) social group, (b) prepandemic risks (time‐lagged variables), and (c) midpandemic circumstances, concerns, and experiences. These models included those who completed a questionnaire at T4 and at T3 or T2 or T1 (*n*, 1055). Following the approach by Pierce et al. (2020), an indicator variable was created (coded as 0 for prepandemic [i.e., T1–T3] and 1 for midpandemic [i.e., T4]) and fitted in all models to capture change in mental health scores between T1–T3 and T4, adjusting for age and the passage of time (i.e., number of days between timepoints). Continuous SDQ scores were used as the outcome in these models to maximise the use of all available data and improve statistical power. As fixed effects regression models quantify the within‐person change in the dependent variable (i.e., SDQ scores), each participant effectively acts as their own control, thereby accounting for potential confounding effects of time‐invariant variables, for example, sex and ethnic group. Positive coefficients indicate worsening – and negative coefficients improving – within‐person mental health between T1–T3 and T4, accounting for prepandemic trends in mental health. To examine variation in the impact of the pandemic on within‐person change in SDQ scores – that is, between‐group differences in within‐person change (i.e., by gender, ethnic group, prepandemic risks, and so on) – we fitted interactions between each independent variable (i.e., social group, pre and midpandemic risks, etc.) and the Covid‐19 indicator (e.g., covidindicator##gender). We then used Stata’s lincom and testparm commands to estimate subgroup‐specific within‐person change in SDQ scores and to examine the strength of evidence for an interaction. The proportions with missing data pre‐ and mid‐Covid were very low (see Appendix S2), so available case analyses, with inverse probability weights, were used. All analyses were completed in Stata Version 16.

In interpreting findings, we focus on the magnitude and precision of estimated effects and, where effects are modest and confidence intervals wide, we are cautious in drawing inferences.

## Results

Between May and August 2020, 1074 completed the T4 questionnaire. Of these, 1055 had completed questionnaires prior to the pandemic (T1–T3) (39% of 2,692 who provided recontact information by May 2020 and 22% of 4,784 who participated at any previous time point). There were some variations in response at T4 by demographic characteristics and prior mental health. Those who completed the T4 questionnaire (vs. those who did not) were more likely to be girls (i.e., 67.5% vs. 46.2%), more likely to be of British White ethnicity (i.e., 21.4% vs. 13.1%), and less likely to be of Black Caribbean ethnicity (9.5% vs. 18.2%). Among boys, but not girls, those with a probable mental health problem (i.e., SDQ scores ≥18) at prior time points, particularly at T2 and T3, were more likely to participate at T4 than those without (Table S1). When applying inverse probability weights, the representativeness of the sample on core demographic variables and prior mental health problems was, broadly, restored. That is, weighted proportions were broadly similar to the REACH total sample (Table S2).

### Social impacts

Overall, social impacts – and related worries and perceived positives – were mixed (Tables S3–S5). For some, home circumstances, relationships, and routines were profoundly disrupted, especially those in low‐income households and in minority ethnic groups (e.g., financial problems at home: minority ethnic groups, 14.9% to 22.1% vs. British White, 10.4%). For others, there were positive changes (e.g., around 31% (*n* 336) reported that relationships with family improved). Almost all reported a mix of concerns and positives related to social restrictions and school closures, with over 50% reporting 4 or more (*n* 583, 53.1%) concerns and 88% reporting 4 or more (*n* 946) positives. The most common concerns included exams and grades (*n* 520, 50.2%), falling behind with schoolwork (*n* 397, 38.3%), and not seeing friends (*n* 345, 33.2%). In general, girls expressed more concerns than boys (i.e., at least one concern: girls *n* 619, 86.4%; boys *n* 246, 70.1%). Concerns related to household financial stability were generally more common among those from Black African and Black Caribbean backgrounds (vs. White British) and among those in receipt of free school meals.

### Mental health (1) Overall

**Figure 1:**
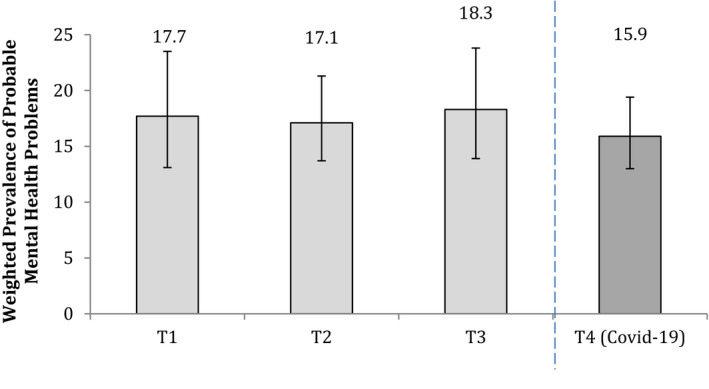
Weighted prevalence of probable mental health problems (SDQ scores ≥18) at each time point

Against a background of high prepandemic levels of mental distress (i.e., 18–20% probable mental health problem), there was no evidence of an overall increase in the prevalence of mental health problems – or in mean SDQ scores or, when modelled longitudinally, within‐person change in distress – pre to midpandemic (Tables 1 and 2 and Figure 1). This was also the case for depression, anxiety, and self‐harm (Table S6 and Figure S1).

**Table 1:** Probable mental health problems (SDQ score ≥18) at each time point

	T1 (2016–17) (*n*, 955)			T2 (2017−18) (*n*, 943)			T3 (2018–19) (*n*, 958)			T4 (Covid‐19) (2020) (*n*, 1,055)		
	*n* ^a^	Weighted %	(95% CI)	*n* ^a^	Weighted %	(95% CI)	*n* ^a^	Weighted %	(95% CI)	*n* ^a^	Weighted %	(95% CI)
Overall	169	**17.7**	(13.1, 23.5)	161	**17.1**	(13.7, 21.3)	170	**18.3**	(13.9, 23.8)	170	**15.9**	(13.0, 19.4)
Sex												
Boys	66	15.0	(9.6, 22.6)	59	13.8	(9.4, 20.0)	61	13.9	(8.1, 23.0)	65	13.3	(9.9, 17.8)
Girls	103	20.0	(14.3, 27.3)	102	19.9	(15.9, 24.6)	109	22.3	(17.4, 27.9)	106	18.1	(15.1, 21.5)
Free school meals												
No	110	15.9	(10.3, 23.7)	110	16.0	(13.1, 19.4)	120	17.8	(12.1, 25.6)	125	16.1	(12.2, 20.8)
Yes	59	22.4	(15.7, 31.0)	51	20.3	(12.8, 30.5)	50	19.7	(11.8, 31.2)	46	15.5	(10.6, 22.0)
Ethnic group												
Black African	44	18.3	(12.2, 26.5)	37	15.0	(10.7, 20.6)	31	12.8	(8.9, 17.9)	35	12.6	(6.5, 23.1)
Black Caribbean	25	18.9	(9.0, 35.4)	23	18.3	(11.3, 28.3)	33	24.9	(10.8, 47.7)	36	24.3	(14.9, 37.2)
British White	25	14.9	(8.8, 24.1)	23	14.6	(8.4, 24.2)	26	16.6	(10.8, 24.7)	31	17.4	(14.5, 20.6)
Non‐British White	17	15.8	(8.8, 26.8)	19	18.4	(10.7, 29.8)	16	17.6	(12.1, 25.0)	16	14.3	(8.7, 22.7)
Mixed	31	21.7	(16.2, 28.3)	28	20.3	(14.8, 27.0)	24	23.5	(14.8, 35.2)	24	14.6	(8.2, 24.5)
Other	27	16.4	(9.3, 27.2)	30	18.5	(12.3, 27.0)	19	19.0	(12.1, 28.5)	28	14.9	(9.6, 22.5)

Percentages are weighted. Robust standard errors are used to account for the clustering of pupils within schools. T, Time.

^a^ Number calibrated to weights and rounded to the nearest whole number.

**Table 2:** Fixed effects regression models: within‐person change pre‐Covid to mid‐Covid, adjusted for age and passage of time (number of days between time points)

	SDQ total difficulties score			SDQ internalising score			SDQ externalising score		
	*b*	95% CI	*p* (interaction)	*b*	95% CI	*p* (interaction)	*b*	95% CI	*p* (interaction)
Overall	−0.06	(−0.66, 0.54)		0.04	(−0.31, 0.39)		−0.27	(−0.63, 0.10)	
*Demographic and pre‐Covid Risks*									
Sex									
Boys	−0.59	(−1.37, 0.19)	.**007**	−0.13	(−0.57, 0.31)	.**131**	−0.61	(−1.07, −0.16)	.**004**
Girls	0.42	(−0.19, 1.03)		0.20	(−0.18, 0.58)		0.04	(−0.35, 0.43)	
Free school meals									
No	0.02	(−0.57, 0.62)	.394	0.03	(−0.32, 0.38)	.851	−0.16	(−0.53, 0.21)	.**069**
Yes	−0.36	(−1.33, 0.61)		0.08	(−0.50, 0.66)		−0.65	(−1.22, −0.07)	
Ethnic group									
British White	0.28	(−0.55, 0.82)	.564	−0.05	(−0.48, 0.37)	.509	0.19	(−0.27, 0.65)	.**006**
Black African	−0.47	(−1.34, 0.40)		−0.09	(−0.60, 0.42)		−0.51	(−1.02, 0.11)	
Black Caribbean	−0.43	(−1.90, 1.04)		0.63	(−0.20, 1.47)		−1.27	(−2.12, −0.42)	
Non‐British White	0.45	(−0.75, 1.65)		0.16	(−0.52, 0.84)		0.14	(−0.54, 0.83)	
Mixed	0.01	(−0.89, 0.92)		0.22	(−0.33, 0.77)		−0.48	(−1.04, 0.08)	
Other	−0.14	(−1.09, 0.80)		−0.11	(−0.69, 0.46)		−0.19	(−0.76, 0.38)	
Mental health problems (at T3)									
No	0.24	(−0.39, 0.87)	.**002**	0.22	(−0.15, 0.59)	.008	−0.11	(−0.49, 0.27)	.**015**
Yes	−1.04	(−1.88, 0.20)		−0.46	(−0.98, 0.06)		−0.77	(−1.32, −0.22)	
Family affluence									
High	−0.22	(−0.91, 0.48)	.**016**	0.07	(−0.47, 0.33)	.211	−0.34	(−0.76, 0.07)	.**012**
Low, moderate	−1.12	(−1.89, −0.36)		−0.36	(−0.82, 0.11)		−0.90	(−1.37, −0.43)	
*Mid‐Covid risks*									
Financial problems									
No	−0.36	(−0.96, 0.24)	.**008**	−0.10	(−0.45, 0.25)	.**008**	−0.43	(−0.81, −0.06)	.**030**
Yes	1.27	(−0.04, 2.58)		0.82	(0.09, 1.55)		0.31	(−0.44, 1.10)	
Change in family relationships									
Lot worse	5.39	(1.10, 9.69)	.**011**	3.55	(1.22, 5.89)	.**007**	1.83	(−0.37, 4.04)	.075
Little worse	0.66	(−0.35, 1.66)		0.31	(−0.30, 0.92)		0.13	(−0.54, 0.80)	
Same	−0.40	(−1.04, 0.23)		−0.18	(−0.56, 0.20)		−0.38	(−0.77, 0.01)	
Little better	0.04	(−0.73, 0.82)		0.13	(−0.35, 0.61)		−0.24	(−0.72, 0.24)	
Lot better	−1.29	(−2.82, 0.25)		−0.59	(−1.44, −0.25)		−0.95	(−1.81, −0.10)	
Freq. argue with parents									
Never	−0.29	(−1.52, 0.93)	.**001**	−0.09	(−0.70, 0.65)	.222	−0.41	(−1.08, 0.47)	.**001**
Hardly ever	−1.03	(−1.73, −0.32)		−0.32	(−0.75, 0.12)		−0.88	(−1.33, −0.42)	
<Once a week	0.63	(−0.35, 1.61)		0.30	(−0.27, 0.87)		0.15	(−0.40, 0.70)	
>Once a week	0.16	(−0.64, 0.96)		0.11	(−0.40, 0.61)		−0.09	(−0.59, 0.43)	
Most days	0.62	(−0.41, 1.64)		0.16	(−0.38, 0.69)		0.21	(−0.61, 1.04)	
Feel Lonely									
Not at all	−1.28	(−1.98, −0.58)	**<.001**	−0.60	(−1.01, −0.18)	**<.001**	−0.84	(−1.28, −0.41)	**<.001**
Slightly	0.17	(−0.47, 0.81)		0.12	(−0.27, 0.50)		−0.12	(−0.53, 0.29)	
Moderately	1.22	(0.03, 2.40)		0.64	(−0.03, 1.32)		0.40	(−0.29, 1.10)	
Very	1.59	(0.30, 2.88)		1.25	(0.50, 2.01)		0.20	(−0.67, 1.08)	
Stable routine									
Not at all	1.11	(0.05, 2.17)	**<.001**	0.80	(0.21, 1.39)	**<.001**	0.14	(−0.50, 0.78)	.149
A little	0.24	(−0.60, 1.08)		0.25	(−0.22, 0.71)		−0.17	(−0.68, 0.35)	
A moderate amount	−0.38	(−1.13, 0.36)		−0.16	(−0.64, 0.31)		−0.44	(−0.90, 0.03)	
A lot	−1.74	(−2.73, −0.78)		−0.99	(−1.55, −0.42)		−0.86	(−1.57, −0.15)	
A great deal	−1.29	(−2.45, −0.10)		−0.77	(−1.45, −0.08)		−0.61	(−1.30, 0.08)	
Index of negative impacts (count)	0.43*	(0.22, 0.65)	**<.001**	0.24	(0.11, 0.36)	**<.001**	0.19	(0.04, 0.33)	.**011**
Number of substantive concerns	0.16*	(0.08, 0.24)	**<.001**	0.10	(0.05, 0.14)	**<.001**	0.06	(0.01, 0.11)	.**013**
Number of positives	−0.17*	(−0.28, −0.06)	.**002**	−0.09	(−0.15, −0.02)	.**008**	−0.08	(−0.15, −0.02)	.**010**

Estimates weighted and robust standard errors used. Model adjusted for age and days passed since data collection. b, regression coefficient. * Coefficients are interaction terms and indicate the amount by which change in SDQ from pre‐ to mid‐Covid increases or decreases for each additional impact, concern, or positive.

**Figure 2:**
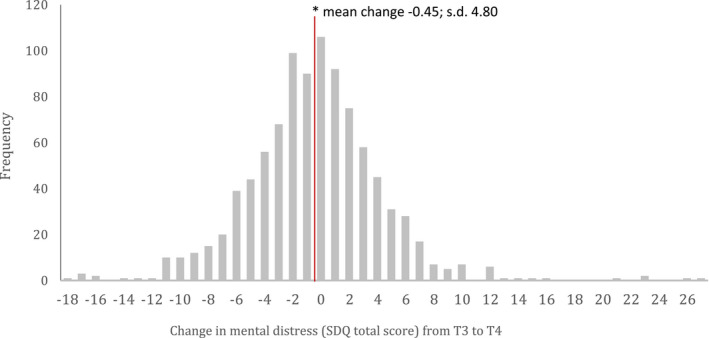
Histogram of crude change in SDQ total difficulties scores between T3 and T4 (mid‐Covid)

However, there was considerable variation around the average of 0 change in SDQ scores, with many reporting marked changes in distress – increases and decreases (see Figure 2, showing the distribution of changes in SDQ scores from T3 to T4, for illustration).

### Mental health (2) Variation in change by demographic group and pre‐Covid‐19 risks

The primary focus in our longitudinal analyses was on modelling variations in within‐person change in distress (SDQ scores) (i.e., interactions) by social group and putative risks to test Hypotheses 2–4 (Table 2 for select variables; Tables S7 and S8 for all variables).

We found some evidence of variations in within‐person change in distress by demographic group. There was a modest variation by gender, with a small increase in distress among girls (0.42 [−0.19, 1.03]), primarily in internalising scores, and a small decrease among boys (−0.59 [−1.25, 0.18]; *p* for interaction, .007), primarily in externalising scores. There were no notable variations in changes in overall distress by ethnic group, but there was some evidence of a decrease in externalising scores among some groups (e.g., Black Caribbean: −1.27 [−2.12, −0.42]; *p* for interaction, .006). Similarly, there was no evidence of variation overall by receipt or not of free school meals, but there was some evidence of a variation in changes in externalising scores, with a decrease among those in receipt of free school meals (−0.65 [−1.22, −0.07]) and no change among those not in receipt of free school meals (−0.16 [−0.53, 0.21]; *p* for interaction .069].

When we examined variation in within‐person change in distress by pre‐Covid‐19 mental health problems and several pre‐Covid‐19 risks, we found: (a) strong evidence of variation by prior mental health problems (i.e., SDQ scores ≥18), with a modest reduction in overall distress, on average, among those with (but not without) prior problems (−1.04 [−1.88, 0.20]; *p* for interaction .002); (b) some evidence for variation by household affluence, with, on average, a small decrease in distress among young people from less affluent (but not more affluent) households prepandemic (−1.12 [−1.89, −0.36]; *p* for interaction .016), mostly reflecting a reduction in externalising scores; (c) no evidence that change in distress, overall or by type, varied notably by the level of other pre‐Covid‐19 risks (e.g., bullying, loneliness, and parental discord). That is, pre‐Covid‐19 disparities in distress linked to household discord, income, and isolation largely persisted or decreased – but did not widen – in the early phase of the pandemic.

### Mental health (3) By mid‐Covid‐19 experiences, worries, and positives

There was stronger evidence of variations in within‐person change in distress by several midpandemic experiences, with – broadly – increases among those reporting negative impacts and decreases among those reporting positive impacts (Table 2 for select variables and Table S9 for all variables). For example, there were notable variations by family relationships, with a marked increase in distress among those who reported that relationships with family were a lot worse than usual (5.39 [1.10, 9.69]) and a decrease among those who reported that relationships were a lot better than usual (−1.29 [−2.82, 0.25]); *p* for interaction, .011). These broad patterns were clearer for internalising than externalising scores. Further, there was strong evidence of variation by household financial circumstances, with evidence of an increase in distress, on average, among those who reported household financial problems at T4 (1.27 [−0.04, 2.58]), but no change among those who did not (−0.36 [−0.96, 0.24]; *p* for interaction, .008). Similar patterns and effects were evident for impacts related to social connections, activities, and routines, that is, around a 1.5 increase in SDQ total scores for the most negative impacts in these domains and around a 1.0 decrease for the most positive impacts.

Further, many young people experienced multiple negative impacts. For example, around 30% (*n* 321) of the cohort reported 2 or more (out of 8) negative impacts and around 10% (*n* 105) 3 or more. Using a simple index counting the number of impacts, we found strong evidence of cumulative effects, such that within‐person increases in distress were amplified with each additional adverse effect. That is, for every additional negative impact, within‐person change in distress increased by around 0.43 [0.22, 0.65].

Finally, we found that the greater the number of concerns the greater the within‐person increase in distress (i.e., for each additional concern an increase, on average, of around 0.16 in SDQ total scores [0.08, 0.24]; *p* < .001) and the greater the number of positives the greater the within‐person decrease in distress (i.e., for each additional positive a decrease, on average, of around 0.17 [−0.24, −0.06]; *p* .002). The magnitude of these effects was similar for both internalising and externalising scores.

## Discussion

This is among the most comprehensive studies of the impacts of Covid‐19 on the mental health of adolescents from diverse ethnic and social backgrounds in a densely populated inner‐city UK sample during the initial period of the pandemic. We found evidence of small variations in changes in mental distress by social group and notable variations in social and economic consequences of the pandemic. Two broad trends emerged. First, mental distress remained high in this cohort and prepandemic disparities in mental distress linked to household discord, income, and isolation largely persisted – but, with some exceptions, did not widen. Second, there was strong evidence of an increase in distress, on average, among those living in challenging circumstances (e.g., financial hardship and poor housing), those directly affected (e.g., worse family relationships, isolation, and unstable routines), and those expressing multiple concerns related to impacts of the pandemic.

### Methodological considerations

Our findings need to be considered in light of several limitations. Most importantly, we were only able to collect information from around 40% (1,074) of the young people we sought to include from the REACH cohort (i.e., 2,692). This was what was practically feasible in the period prior to schools reopening, using online methods. This introduces bias (e.g., due to digital poverty and lack of access to the internet and computers). It is also notable, for example, that the prevalence of lifetime self‐harm at T4 (17%) was slightly lower than at T3 (15%), which may reflect higher attrition among those with higher levels of distress. It is consequently possible that our findings misrepresent the extent of the impacts of the pandemic and underestimate the level of and changes in mental health problems among young people in inner‐London and by social and ethnic group. We did have extensive pre‐Covid‐19 data which allowed us to identify possible sources of bias, analyse predictors of response, and create inverse probability weights, which broadly restored representativeness of the sample on core variables. However, this does not fully address the challenge of potential bias (e.g., due to unmeasured predictors of response) and inverse probability weights may bias (likely overestimate) standard errors (Vamvakas, Norbury, & Pickles, 2021). Caution is therefore still needed in drawing inferences from our findings, especially where effects were modest and given the large number of analyses we conducted.

At T1–T3, all questionnaires were administered in‐class, on study tablets, with trained researchers present to answer questions. At T4, data were collected remotely. In using a remotely completed self‐report questionnaire, the potential for measurement error and misclassification (e.g., in mental health status) is high. This limitation is offset, to some extent, by our use of validated measures that have been used extensively in previous epidemiological studies of adolescent mental health (e.g., the SDQ). This limitation characterises all Covid‐19 research, given the restrictions on face‐to‐face interviews.

In addition, T1–T3 data were collected throughout the academic year (September–July). The T4 data included in this analysis were collected during a 4‐month window (May–August 2020). We restricted analyses to this time window because of the importance of producing analyses relevant to the initial stages, and short‐term impacts, of the pandemic. This approach was reinforced by feedback from our Young Person Advisory Group, which stressed the importance of capturing impacts at different stages of the pandemic. This noted, it is possible that restriction to this period impacts comparisons with data from T1 to T3. To mitigate this, all regression models were adjusted for the passage of time. Further, our main outcome measure, the SDQ, covers a 6‐month period. We would not, therefore, expect this measure of distress – and its ability to capture distress during the period of lockdown – to be impacted by the precise timing of assessment within a 4‐month window of data collection.

There are limitations to using predefined cut‐points to indicate high levels of distress, particularly when applying such thresholds to wide age ranges and diverse samples. For example, the SDQ thresholds have been validated for 11–17‐year‐olds, but not – as far as we are aware – for 18‐year‐olds. In our analyses, we used the established cut‐points for 11–17‐year‐olds because only 8 participants (<1%) were aged 18 at T4, so it is unlikely that the use of alternative thresholds would have substantively altered overall prevalence estimates. Further, there is some support for (full or partial) measurement invariance of the adolescent self‐report SDQ across genders, ages, and ethnic groups in several European countries (Bøe, Hysing, Skogen, & Breivik, 2016; Goodman, Patel, & Leon, 2010; Van Roy, Veenstra, & Clench‐Aas, 2008). However, the evidence is not consistent (e.g., Richter, Sagatun, Heyerdahl, Oppedal, & Røysamb, 2011; Yao et al., 2009) and the validity of pre‐specified thresholds to identify high levels of distress across all groups consequently remains somewhat uncertain. Similarly, the thresholds used to indicate the presence of anxiety on the GAD‐7 were developed in older age groups (18+) (Spitzer et al., 2006) and their appropriateness for younger groups is not clear. It is for these reasons that we focussed our analyses, beyond Hypothesis 1, on continuous SDQ scores. Specifically, we used total difficulties, internalising, and externalising scores, as recommended for community‐based samples (Goodman, Lamping, et al., 2010; Mieloo et al., 2014; Richter et al., 2011). In relation to Hypothesis 1, our findings were consistent across a range of measures and for both categorical and continuous scores.

For cross‐sectional measures at T4 of impacts and experiences related to the pandemic and social restrictions, we cannot rule out the possibility of reverse effects; it is possible that increases in mental distress occurred prior to – and therefore influenced – reports of concerns and worries. However, given that most worries related to challenges that arose because of the pandemic, this seems implausible and it is more difficult still to argue that increases in young people’s mental distress influenced housing problems, household financial problems, and so on. Further, those who were already distressed may have been more inclined to appraise their circumstances negatively, a possibility made more likely by our use of single informants. That said, our use of the same measure and informant across time points meant we were able to assess changes in levels of distress over time, independent of measurement differences that may arise in the use of multiple informants. Finally, it is possible that some findings, for example, modest reductions in distress among those with prior mental health problems, simply reflect regression to the mean.

There are several notable strengths of REACH. For example, it comprises a diverse inner‐city sample, with large proportions from minority ethnic groups and disadvantaged backgrounds, groups often under‐represented in other cohorts. Studies based on national samples may obscure important variations by place and clustered adversities to which some social and ethnic groups are exposed. Indeed, rates of infection, morbidity, and mortality due to Covid‐19 have varied widely across the United Kingdom, and by social and ethnic groups. The geographical focus of REACH may limit the extent to which we can generalise to other regions and nationally, but it provides much‐needed data about impacts among a more diverse and disadvantaged population. Further, data were collected at multiple and regular time points, including immediately prepandemic, which meant we were able to model within‐person change in distress longitudinally – the first study to date in this age group, as far as we are aware, to do so.

### Trends in mental distress pre‐ and mid‐Covid‐19

In line with several other reports, we found that the initial impacts of social restrictions and school closures on daily routines, relationships, and education – and related concerns and perceived positives – among young people in inner‐London were mixed (Ellis, Dumas, & Forbes, 2020; Hawke et al., 2020; Magson et al., 2021; Widnall et al., 2020). It is perhaps not surprising, given this, that we found no evidence of an overall increase in mental distress. These findings broadly align with other, but not all (Newlove‐Delgado et al., 2021), similarly designed longitudinal studies that have assessed and compared levels of mental distress prepandemic and midpandemic (Patalay & Fitzsimons, 2020; Widnall et al., 2020). With the exception of the MHCYP (Newlove‐Delgado et al., 2021), most studies that report high levels of mental distress among young people midpandemic used different designs, that is of convenience samples, recruited online, with no pre‐Covid‐19 data for comparison (e.g. Young Levita et al., 2020; Minds, 2020). It is possible, then, that where findings differ between our and other studies it is, in part, because of differences in design. In short, the most robust studies, with pre‐Covid data, and that sought to minimise biases due to attrition, are consistent with ours (e.g., no strong evidence of an increase in mental distress).

However, these overall trends mask inequalities; some were clearly impacted more than others. For example, we found a modest increase in distress, mostly internalising problems, among girls, such that prepandemic disparities between girls and boys widened. This is in line with some other reports (Hawes, Szenczy, Klein, Hajcak, & Nelson, 2021; Patalay & Fitzsimons, 2020). It is plausible that school closures and social restrictions have affected boys and girls differently, and those in poorer and more marginalised groups. It may be, for instance, that the areas of life most disrupted – that is, education, peer relationships, family health – are more salient concerns for girls and therefore a greater source of worry.

Further, there were some groups that reported, on average, decreases in forms of mental distress. For example, contrary to expectations, we found evidence of a decrease in externalising problems among some ethnic groups (e.g., Black Caribbean) and those in low‐income households. It may be that these changes reflect normal fluctuations over time. But it is also possible that prior problems were linked to more challenging experiences at school (e.g. peer pressures; academic pressures) for some groups and for some from low‐income households, and that – initially at least – a period away from school was beneficial. This may also explain our finding that those with mental health problems prior to the pandemic experienced, on average, a decrease in mental distress. It is notable that Widnall et al. (2020) – in the only other study we are aware of with relevant data in the 6–12 months immediately pre and midpandemic – also found a decrease on average in mental distress among those who reported poor mental health prepandemic (Widnall et al., 2020).

### Social impacts of Covid‐19 and mental distress

The strongest impacts on mental health – both negative and positive – were evident for those variables that captured changes in circumstances and relationships consequent on the pandemic and social restrictions. For example, we found an increase in mental distress among young people who reported challenging household circumstances (e.g., financial and housing problems) and negative impacts on social connections, activities, and routines. Several other studies have reported similar findings (Magson et al., 2021; Vizard et al., 2020). For example, in the UK Mental Health of Children and Young People Survey, children (age 5–16 years) with a probable mental disorder (vs. those without) were more likely to live in households that had fallen behind with the payment of bills (16.3% vs. 6.4%), less likely to spend time with family (7.4% vs. 1.3%), and less likely to do physical exercise (15.1% vs. 5.5%) (Vizard et al., 2020). Our study strengthens these emerging findings by modelling associations between negative impacts and within‐person change in distress over time, controlling for potential confounding by time‐invariant factors, and enabling stronger causal inferences.

### Implications

Adolescence is an important development stage, during which interconnected biological, psychological, and social processes have long‐lasting impacts on subsequent education, work, relationships, and health. Understanding the varying impacts of the Covid‐19 pandemic and social restrictions on young people’s mental health is consequently important in developing appropriate responses to mitigate these in the most affected groups. Several implications follow.

First, the positive experiences that many young people reported, and the benefits of these for their mental health, cast light on aspects of young people’s lives that, prepandemic, were sources of stress, anxiety, and unhappiness. These may, in turn, provide valuable pointers for social and education policy as attention shifts to rebuilding society postpandemic. Our findings that the mental health of some in more marginalised and vulnerable groups improved with the closure of schools suggests that, unintentionally, this afforded some protections against harmful expectations and relationships and, as such, reflects poorly on our current education systems. This suggests that more consideration should be given to how we can better support those who find school challenging and, more boldly, to how the education systems can be restructured to mitigate inequalities.

Second, the most frequently reported concerns related to education – to concerns about exams, falling behind with schoolwork, and advancing to further education. This is in line with findings from other surveys that suggest the impact of the pandemic on education was a prominent factor in increasing levels of distress among young people (Mansfield et al., 2021; Vizard et al., 2020; Young Minds, 2020). Together, these findings emphasise the importance of mitigating impacts on schooling by, for example, prioritising in‐school teaching, providing necessary resources (e.g., computer and internet access) for online learning, and ensuring certainty around format and processes for exams and for grading.

Third, the impacts were greatest among those who reported financial hardship, poor housing, worse relationships and isolation, and disruption to routines. Government income and other schemes in the United Kingdom (and elsewhere) have, undoubtedly, mitigated the impacts of social restrictions and economic recession on the mental health of families and their children, by providing a measure of stability at a time of considerable worry and threat. As such, they point to the potential longer‐term benefits of strategies to prevent household poverty and insecurity for the mental health of young people. Further, irrespective of overall changes in mental distress, young people living in households directly impacted by the economic and social consequences of the pandemic are more at risk. Consideration should be given to how these young people can be identified and supported – in non‐stigmatising ways – by schools and communities to prevent initial, understandable distress from crystallising into long‐term, more intractable disorders.

In sum, responding to the impacts of the pandemic on the mental health of young people requires social and economic policy, public health strategies, and community‐based and school‐wide interventions.

Finally, our data provide insights that relate to the first period of lockdown in the United Kingdom and elsewhere and it may be that impacts have changed over time. We plan further waves of data collection to capture these impacts. Future research is essential to develop the evidence base to inform effective responses to limit the long‐term negative effects of the pandemic on the lives of a generation of young people.

## Funding

REACH is supported by funding from the European Research Council (Ref: REACH 648837), the ESRC (ESRC Centre for Society and Mental Health at King’s College London: ESRC Reference: ES/S012567/1), and UKRI (Ref: COV0491). S.L.H. and C.M. are part‐funded by the NIHR Biomedical Research Centre at South London and Maudsley NHS Foundation Trust and King’s College London. The funders had no role in study design, data collection, data analysis, data interpretation, or writing of the report.

## Data access

C.M. had full access to all the data in the study and takes responsibility for the integrity of the data and the accuracy of the data analysis.

**Key points**

- There is widespread concern about the impacts of Covid‐19 and related social restrictions on young people’s mental health.
- The evidence so far is mixed; however, most studies are based on convenience samples which are limited by selection bias, lack of pre‐Covid‐19 data, and underrepresentation of young people from diverse groups.
- We analysed four waves of data on mental health – 3 pre‐Covid‐19 and 1 mid‐Covid‐19 – from our cohort study of young people from diverse ethnic and social backgrounds (>80% minority ethnic groups), REACH.
- We found no evidence for an overall increase in mental distress, but we found notable variations in distress by impacts of Covid‐19.
- The strongest evidence for increases in mental distress was among those living in challenging circumstances and those most directly affected by social restrictions.

## Supporting information


**Appendix S1**. T4 Questionnaire.
**Appendix S2**. Missing Data.
**Table S1**. Comparing those who completed T4 questionnaire with those who did not
**Table S2**. Sample characteristics of T4 sample (actual, weighted) compared with full REACH cohort and target population
**Table S3**. Social circumstances, relationships, and routines mid‐covid‐19 (note: frequencies and percentages are descriptive, not weighted)
**Table S4**. Reported worries or concerns (note: frequencies and percentages are descriptive, not weighted).
**Table S5**. Reported positives (note: frequencies and percentages are descriptive, not weighted).
**Table S6**. Weighted prevalence estimates and 95% confidence intervals of depression, anxiety, and lifetime self‐harm pre‐ and mid‐covid‐19
**Table S7**. Social circumstances and experiences pre‐covid‐19 (note: frequencies and percentages are descriptive, not weighted).
**Table S8**. Fixed effects regression models: within‐person change pre‐covid to mid‐covid, overall, and by demographic group and select pre‐Covid‐19 risks, adjusted for age and passage of time (number of days between timepoints)
**Table S9**. Fixed effects regression models: within‐person change pre‐covid to mid‐covid, by mid‐Covid‐19 circumstances, experiences, and routines, adjusted for age and passage of time (number of days between timepoints)
**Figure S1**. Weighted prevalence estimates and 95% confidence intervals of depression, anxiety, and lifetime self‐harm at each time point.
