# Supplementary material for: Covid‐19, social restrictions, and mental distress among young people: a UK longitudinal, population‐based study
Source: J Child Psychol Psychiatry. 2022 Feb 23:10.1111/jcpp.13586. Online ahead of print. doi: 10.1111/jcpp.13586 (PMC9114888; doi:10.1111/jcpp.13586)
Supplement: Supplementary file 1 — Appendix S1. T4 Questionnaire. Appendix S2. Missing Data. Table S1. Comparing those who completed T4 questionnaire with those who did not Table S2. Sample characteristics of T4 sample (actual, weighted) compared with full REACH cohort and target population Table S3. Social circumstances, relationships, and routines mid‐covid‐19 (note: frequencies and percentages are descriptive, not weighted) Table S4. Reported worries or concerns (note: frequencies and percentages are descriptive, not weighted). Table S5. Reported positives (note: frequencies and percentages are descriptive, not weighted). Table S6. Weighted prevalence estimates and 95% confidence intervals of depression, anxiety, and lifetime self‐harm pre‐ and mid‐covid‐19 Table S7. Social circumstances and experiences pre‐covid‐19 (note: frequencies and percentages are descriptive, not weighted). Table S8. Fixed effects regression models: within‐person change pre‐covid to mid‐covid, overall, and by demographic group and select pre‐Covid‐19 risks, adjusted for age and passage of time (number of days between timepoints) Table S9. Fixed effects regression models: within‐person change pre‐covid to mid‐covid, by mid‐Covid‐19 circumstances, experiences, and routines, adjusted for age and passage of time (number of days between timepoints) Figure S1. Weighted prevalence estimates and 95% confidence intervals of depression, anxiety, and lifetime self‐harm at each time point. [file JCPP-9999-0-s001.docx]

**Supporting Information**

**Appendix S1. T4 Questionnaire**

**Mental health (T1 to T4)**

Overall and specific types of mental distress was assessed at T1-T4 using age-appropriate, widely used, validated self-report measures:

1. Strengths and Difficulties Questionnaire (SDQ):^1,2^ a 25-item measure of emotional and behavioural difficulties during the previous 6 months. Items are rated on a 3-point scale and span five subdomains: emotional problems, conduct problems, hyperactivity-inattention, peer problems, and prosocial behaviours. Following established procedures, we calculated a total difficulties score (0 to 40) (TDS) and internalising and externalising scores (0 to 20). Higher scores indicate more difficulties. TDS scores ≥18 indicate probable mental health problems. The SDQ has been shown to have satisfactory internal consistency,^3^ and good concurrent and discriminant validity.^4,5^
2. Short Mood and Feelings Questionnaire (SFMQ):^6,7^ a 13-item measure of depression in the past 2 weeks. Items are rated on a 3-point scale and scores ≥12 indicate high risk of depression. The SMFQ has high internal consistency and convergent validity, and moderate diagnostic accuracy, among adolescents.^6,7^
3. The Generalised Anxiety Disorder Scale (GAD-7):^8,9^ a 7-item measure of anxiety in the past 2 weeks. Items are rated on a 4-point scale and scores ≥10 indicate moderate-to-severe anxiety. Recent community-based research supports the validity and reliability of the GAD-7 among adolescents.^9^
4. Lifetime self-harm: A single item on lifetime self-harm from the Development and Adolescent Wellbeing Assessment.^10^

**Impacts and experiences of Covid-19 and related restrictions**

We reviewed all emerging national and international Covid-19-related mental health research to identify measures of impacts and experiences to maximise data comparability. We included:

**Covid-19** **infection** Three items on whether the participant, anyone in their household, or another family member had or was suspected of having Covid-19 [Co-SPACE].^11^

**Housing** A 10-item version of the housing quality scale from Understanding Society^12^ to assess quality of living space (e.g., damp, rot); and items on access to private outdoor space (e.g., garden, balcony) [Co-SPACE],^11^ own bedroom, own computer or tablet, and access to internet (Family Affluence Scale).^13^

**Economic impacts** 8 items derived from the Covid-19 Adolescent Symptom & Psychological Experience Questionnaire (CASPE)^14^ comprising: job loss of one parent; job loss of both parents; decrease in household income; difficulty paying rent, bills, or buying necessities; adult working longer hours; family applying for welfare benefits; first use of food bank; and family evicted or lost accommodation; 1 item from the Adolescent-appropriate Life Events Checklist^15,16^ on self-reported financial problems in the past year.

**Social relationships and supports** Items on perceived change in quality of relationships with family [CRISIS];^17^ frequency of arguments with parent(s)/carer(s); availability of peer and adult confidantes; and perceived loneliness.

**Lifestyle and daily routine** Two items on sleep duration and difficulties sleeping [CRISIS];^17^ two items on number of days in the past week engaged in at least 30 minutes of moderate-to-vigorous physical activity (MVPA) [UCL Covid-19 Social Study]^18^ and change in MVPA due to pandemic [CRISIS];^17^ and an item on extent to which participants felt they had a stable daily routine in the last four weeks [The Pandemic Project].^19^

**Worries, concerns, and positives** Twenty-five items on perceived pandemic-related worries, taken from the CASPE^14^ and the UCL Covid-19 Social Study,^18^ and revised following conversations with our Young Persons Advisory Group (YPAG). Similarly, fourteen items on perceived positive consequences of the pandemic in last 4 weeks were also included in the study, derived from the CASPE,^14^ and modified according to feedback from our YPAG.

**Ethnic group** Ethnic group was self-reported by participants at T1-T3 using the ethnic group question from the 2011 UK Census, which has 18 response options. In this analysis we used 6 ethnic group categories: Black African, Black Caribbean, British White, non-British White, mixed, and other. Four of these are original response options: Black African, Black Caribbean, British White, and non-British White. The ‘mixed’ group is a combination of those who identified as ‘mixed black and white’ or ‘other mixed’. The ‘other’ group combines those who self-identified as ‘other’ based on the original response options, plus those in smaller ethnic groups, such as ‘Chinese’ and ‘Arab’, which - due to very small group sizes - could not be included as distinct groups.

**References**

1. Goodman A, Lamping DL, Ploubidis GB. When to use broader internalising and externalising subscales instead of the hypothesised five subscales on the Strengths and Difficulties Questionnaire (SDQ): data from British parents, teachers and children. *Journal of abnormal child psychology* 2010; **38**(8): 1179-91.

2. Goodman R, Meltzer H, Bailey V. The Strengths and Difficulties Questionnaire: a pilot study on the validity of the self-report version. *European child & adolescent psychiatry* 1998; **7**(3): 125-30.

3. Goodman R. Psychometric properties of the strengths and difficulties questionnaire. *Journal of the American Academy of Child and Adolescent Psychiatry* 2001; **40**(11): 1337-45.

4. Lundh LG, Wangby-Lundh M, Bjarehed J. Self reported emotional and behavioral problems in Swedish 14 to 15-year-old adolescents: A study with the self-report version of the Strengths and Difficulties Questionnaire. *Scandinavian Journal of Psychology* 2008; **49**: 523–32.

5. Muris P, Meesters C, van den Berg F. The Strengths and Difficulties Questionnaire (SDQ): Further evidence for its reliability and validity in a community sample of Dutch children and adolescents. *European Child and Adolescent Psychiatry* 2003; **12**: 1–8.

6. Ancold A, Stephen C. Development of a short questionnaire for use in epidemiological studies of depression in children and adolescents. *Age (years)* 1995; **6**(11): 237-49.

7. Thabrew H, Stasiak K, Bavin LM, Frampton C, Merry S. Validation of the mood and feelings questionnaire (mfq) and short mood and feelings questionnaire (smfq) in new zealand help‐seeking adolescents. *International Journal of Methods in Psychiatric Research* 2018; **27**(3): e1610.

8. Spitzer RL, Kroenke K, Williams JB, Löwe B. A brief measure for assessing generalized anxiety disorder: the GAD-7. *Archives of internal medicine* 2006; **166**(10): 1092-7.

9. Tiirikainen K, Haravuori H, Ranta K, Kaltiala-Heino R, Marttunen M. Psychometric properties of the 7-item Generalized Anxiety Disorder Scale (GAD-7) in a large representative sample of Finnish adolescents. *Psychiatry research* 2019; **272**: 30-5.

10. Goodman R, Ford T, Richards H, Gatward R, Meltzer H. The development and well‐being assessment: Description and initial validation of an integrated assessment of child and adolescent psychopathology. *Journal of child psychology and psychiatry* 2000; **41**(5): 645-55.

11. Cresswell C. COVID-19: Supporting Parents, Adolescents and Children during Epidemics (The Co-SPACE Study). 2020.

12. Buck N, McFall S. Understanding Society: design overview. *Longitudinal and Life Course Studies* 2011; **3**(1): 5-17.

13. Wardle J, Robb K, Johnson F. Assessing socioeconomic status in adolescents: the validity of a home affluence scale. *Journal of Epidemiology & Community Health* 2002; **56**(8): 595-9.

14. Ladouceur C. COVID-19 Adolescent Symptom & Psychological Experience (CASPE) Questionnaire. 2020.

15. Cullen AE, Fisher HL, Roberts RE, Pariante CM, Laurens KR. Daily stressors and negative life events in children at elevated risk of developing schizophrenia. *The British Journal of Psychiatry* 2014; **204**(5): 354-60.

16. Heubeck B, O'Sullivan C. An exploration into the nature, frequency and impact of school hassles in the middle school years. *Australian Psychologist* 1998; **33**(2): 130-7.

17. Nikolaidis A, Paksarian D, Alexander L, et al. The Coronavirus Health and Impact Survey (CRISIS) reveals reproducible correlates of pandemic-related mood states across the Atlantic. *medRxiv* 2020: 2020.08.24.20181123.

18. Fancourt D, Bu F, Mak HW, Steptoe A. COVID-19 social study. *Results release* 2020; **15**.

19. Pennebaker JW. Pandemic Project. 2020.

**Appendix S2. Missing Data**

For demographics and the SDQ, our primary mental health outcome, the proportions with missing data were <5%. For example, ethnic group and gender were missing for 15 (1.4%) participants, year group was missing for 35 (3.3%), pre-covid SDQ scores were missing for 7 (0.7%), and mid-covid SDQ scores were missing for 2 (0.2%). For covid-related (T4) experiences, perceptions, and circumstances, missingness was also low: e.g., <1% (change in family relationships, own bedroom, covid-19 infection, change in household financial income, peer and adult confidantes); 3-4% (concerns and positives related to lockdown); or 5-7% (keyworker in the household, parental discord, financial problems at home, parental drinking problems), with the exception of parental occupation, which was missing for 192 (17.9%) participants who did not know their parents’/carers’ occupational class.

**Table S1**. Comparing those who completed T4 questionnaire with those who did not.

|  | | **Participated at T4**  **(n, 1074)** | | **Did not participate at T4 (n, 3,710)** | |  |  |  |
| --- | --- | --- | --- | --- | --- | --- | --- | --- |
|  | | **n** | **%** | **n** | **%** | **X^2^** | **df** | **p** |
| Sex | |  |  |  |  |  |  |  |
|  | Boys | 349 | 32.5 | 1,995 | 53.8 | 151.10 | 1 | <0.001 |
|  | Girls | 725 | 67.5 | 1,714 | 46.2 |  |  |  |
| Free school meals | |  |  |  |  |  |  |  |
|  | No | 792 | 74.8 | 2,569 | 70.6 | 7.20 | 1 | 0.007 |
|  | Yes | 267 | 25.2 | 1,072 | 29.4 |  |  |  |
| Ethnic group | |  |  |  |  |  |  |  |
|  | Black African | 281 | 26.4 | 940 | 25.5 | 77.86 | 5 | <0.001 |
|  | Black Caribbean | 101 | 9.5 | 673 | 18.2 |  |  |  |
|  | Mixed | 160 | 15.0 | 535 | 14.5 |  |  |  |
|  | British White | 228 | 21.4 | 485 | 13.1 |  |  |  |
|  | Non-British White | 105 | 9.9 | 352 | 9.5 |  |  |  |
|  | Other | 190 | 17.8 | 707 | 19.2 |  |  |  |
| Cohort | |  |  |  |  |  |  |  |
|  | 1 | 439 | 40.9 | 1,331 | 35.9 | 19.80 | 2 | <0.001 |
|  | 2 | 362 | 33.7 | 1,177 | 31.7 |  |  |  |
|  | 3 | 273 | 25.4 | 1,202 | 32.4 |  |  |  |
| Probable mental health problem | |  |  |  |  |  |  |  |
|  | T1: Overall | 188 | 19.5 | 618 | 18.5 | 0.53 | 1 | 0.464 |
|  | T1: Boys | 55 | 17.3 | 276 | 15.6 | 0.74 | 1 | 0.388 |
|  | T1: Girls | 133 | 20.7 | 342 | 21.9 | 0.43 | 1 | 0.510 |
|  | T2: Overall | 182 | 19.2 | 480 | 15.9 | 6.09 | 1 | 0.014 |
|  | T2: Boys | 55 | 18.0 | 187 | 11.7 | 9.42 | 1 | 0.002 |
|  | T2: Girls | 127 | 19.8 | 293 | 20.5 | 0.12 | 1 | 0.730 |
|  | T3: Overall | 182 | 18.9 | 437 | 17.3 | 1.20 | 1 | 0.274 |
|  | T3: Boys | 53 | 16.6 | 160 | 11.9 | 5.14 | 1 | 0.023 |
|  | T3: Girls | 129 | 20.0 | 277 | 23.6 | 2.99 | 1 | 0.084 |

**Table S2.** Sample characteristics of T4 sample (actual, weighted) compared with full REACH cohort and target population.

|  | | **T4 sample**  (n, 1074) | | **T4 sample, weighted**  (n, 1074) | | **REACH total sample**  (n, 4353) | | **Target Population* at T1** |
| --- | --- | --- | --- | --- | --- | --- | --- | --- |
|  | | **n** | **%** | **n, calibrated** | **%** | **n** | **%** | **%** |
| Sex | |  |  |  |  |  |  |  |
|  | Boys | 349 | 32.5 | 488 | 45.4 | 2,138 | 49.1 | 50.5 |
|  | Girls | 725 | 67.5 | 586 | 54.6 | 2,215 | 50.9 | 49.5 |
| Free school meals | |  |  |  |  |  |  |  |
|  | No | 792 | 74.8 | 779 | 72.6 | 3,137 | 76.3 | 74.8 |
|  | Yes | 267 | 25.2 | 295 | 27.4 | 976 | 23.7 | 25.2** |
| Ethnic group | |  |  |  |  |  |  |  |
|  | Black African | 281 | 26.4 | 279 | 26.0 | 1,113 | 25.6 | 27.2 |
|  | Black Caribbean | 101 | 9.5 | 146 | 13.6 | 719 | 16.5 | 14.0 |
|  | Mixed | 160 | 15.0 | 167 | 15.5 | 617 | 14.2 | 12.5 |
|  | British White | 228 | 21.4 | 180 | 16.7 | 667 | 15.3 | 16.4 |
|  | Non-British White | 105 | 9.9 | 113 | 10.5 | 626 | 14.4 | 10.8 |
|  | Other | 190 | 17.8 | 190 | 17.7 | 1243 | 28.6 | 23.3 |
| School year at T4 | |  |  |  |  |  |  |  |
|  | Year 8 | 44 | 4.1 | 28 | 2.6 | - | - | - |
|  | Year 9 | 122 | 11.4 | 129 | 12.0 | - | - | - |
|  | Year 10 | 262 | 24.4 | 240 | 22.3 | - | - | - |
|  | Year 11 | 387 | 36.0 | 385 | 35.9 | - | - | - |
|  | Year 12 - 13 | 259 | 24.1 | 292 | 27.2 | - | - | - |
| Probable mental health problem | |  |  |  |  |  |  |  |
|  | T1: Overall | 188 | 19.5 | 169 | 17.7 | 806 | 18.8 | - |
|  | T1: Boys | 55 | 17.3 | 66 | 15.0 | 331 | 15.9 | - |
|  | T1: Girls | 133 | 20.7 | 103 | 17.3 | 475 | 21.5 | - |
|  | T2: Overall | 182 | 19.2 | 160 | 17.1 | 662 | 16.7 | - |
|  | T2: Boys | 55 | 18.0 | 59 | 13.8 | 242 | 12.7 | - |
|  | T2: Girls | 127 | 19.8 | 102 | 19.9 | 420 | 20.3 | - |
|  | T3: Overall | 182 | 18.9 | 170 | 18.3 | 619 | 17.8 | - |
|  | T3: Boys | 53 | 16.6 | 61 | 13.9 | 213 | 12.8 | - |
|  | T3: Girls | 129 | 20.0 | 109 | 22.2 | 406 | 22.4 | - |

T1, Time 1 (2016/17). T2, Time 2 (2017/2018). T3, Time 3 (2018/2019). T4, Time 4 (May 2020-Aug 2020). *Lambeth and Southwark Key Stage 3 pupil demographics obtained from the National Pupil Database Spring 2017 School Census. **Free school meals data for Lambeth and Southwark is not available by Key Stage so the data presented here (percentage of Lambeth and Southwark pupils receiving free school meals) is for Key Stage 3 and Key Stage 4 pupils combined (25.2%, 2017 Spring Census. Source: Department for Education).

**Table S3.** Social circumstances, relationships, and routines mid-covid-19 (note: frequencies and percentages are descriptive, not weighted).

|  | | **Total** | | **Sex** | | | | **Free School Meals** | | | | **Ethnic Group** | | | | | | | | | | | |
| --- | --- | --- | --- | --- | --- | --- | --- | --- | --- | --- | --- | --- | --- | --- | --- | --- | --- | --- | --- | --- | --- | --- | --- |
|  |  | **(n, 1074)** | | **Boys  (n, 348)** | | **Girls (n, 720)** | | **No (n, 843)** | | **Yes (n, 216)** | | **B. African (n, 279)** | | **B. Caribbean (n, 101)** | | **Br. White (n, 238)** | | **non-Br. W. (n, 102)** | | **Mixed (n, 155)** | | **Other (n, 184)** | |
|  | | **n** | **%** | **n** | **%** | **n** | **%** | **n** | **%** | **n** | **%** | **n** | **%** | **n** | **%** | **n** | **%** | **n** | **%** | **n** | **%** | **n** | **%** |
| **Covid-19 infection** | |  |  |  |  |  |  |  |  |  |  |  |  |  |  |  |  |  |  |  |  |  |  |
| Self | |  |  |  |  |  |  |  |  |  |  |  |  |  |  |  |  |  |  |  |  |  |  |
|  | No | 822 | 86.7 | 263 | 85.9 | 556 | 87.1 | 642 | 85.6 | 168 | 90.8 | 223 | 89.6 | 80 | 89.9 | 178 | 81.3 | 76 | 85.4 | 113 | 84.3 | 143 | 91.1 |
|  | Yes | 126 | 13.3 | 43 | 14.1 | 82 | 12.9 | 108 | 14.4 | 17 | 9.2 | 26 | 10.4 | 9 | 10.1 | 41 | 18.7 | 13 | 14.6 | 21 | 15.7 | 14 | 8.9 |
| Family at home | |  |  |  |  |  |  |  |  |  |  |  |  |  |  |  |  |  |  |  |  |  |  |
|  | No | 839 | 79.6 | 278 | 81.0 | 557 | 78.9 | 656 | 78.8 | 171 | 82.2 | 226 | 82.5 | 84 | 84.8 | 172 | 72.6 | 75 | 75.8 | 119 | 78.8 | 151 | 83.9 |
|  | Yes | 215 | 20.4 | 65 | 19.0 | 149 | 21.1 | 176 | 21.2 | 37 | 17.8 | 48 | 17.5 | 15 | 15.2 | 65 | 27.4 | 24 | 24.2 | 32 | 21.2 | 29 | 16.1 |
| Other family | |  |  |  |  |  |  |  |  |  |  |  |  |  |  |  |  |  |  |  |  |  |  |
|  | No | 762 | 72.3 | 258 | 75.2 | 500 | 70.8 | 590 | 70.9 | 159 | 76.4 | 225 | 82.1 | 69 | 69.7 | 155 | 65.4 | 72 | 72.7 | 108 | 71.5 | 123 | 68.3 |
|  | Yes | 292 | 27.7 | 85 | 24.8 | 206 | 29.2 | 242 | 29.1 | 49 | 23.6 | 49 | 17.9 | 30 | 30.3 | 82 | 34.6 | 27 | 27.3 | 43 | 28.5 | 57 | 31.7 |
| **Household** | |  |  |  |  |  |  |  |  |  |  |  |  |  |  |  |  |  |  |  |  |  |  |
| Change in income | |  |  |  |  |  |  |  |  |  |  |  |  |  |  |  |  |  |  |  |  |  |  |
|  | 0 | 692 | 64.4 | 228 | 65.5 | 461 | 64.0 | 539 | 63.9 | 143 | 66.2 | 188 | 67.4 | 65 | 64.4 | 151 | 63.4 | 67 | 65.7 | 96 | 61.9 | 116 | 63.0 |
|  | 1 | 271 | 25.2 | 83 | 23.9 | 187 | 26.0 | 222 | 26.3 | 47 | 21.8 | 67 | 24.0 | 26 | 25.7 | 63 | 26.5 | 26 | 25.5 | 46 | 29.7 | 41 | 22.3 |
|  | 2+ | 111 | 10.3 | 37 | 10.7 | 72 | 9.9 | 82 | 9.7 | 26 | 12.1 | 24 | 8.7 | 10 | 9.9 | 24 | 10.1 | 9 | 8.9 | 13 | 8.4 | 27 | 14.7 |
| Financial problems | |  |  |  |  |  |  |  |  |  |  |  |  |  |  |  |  |  |  |  |  |  |  |
|  | No | 855 | 84.4 | 275 | 84.6 | 577 | 84.4 | 689 | 86.0 | 154 | 77.4 | 226 | 85.0 | 74 | 77.9 | 206 | 89.6 | 80 | 85.1 | 121 | 84.0 | 138 | 80.2 |
|  | Yes | 158 | 15.6 | 50 | 15.4 | 107 | 15.6 | 112 | 14.0 | 45 | 22.6 | 40 | 15.0 | 21 | 22.1 | 24 | 10.4 | 14 | 14.9 | 23 | 16.0 | 34 | 19.8 |
| Parents often argue | |  |  |  |  |  |  |  |  |  |  |  |  |  |  |  |  |  |  |  |  |  |  |
|  | No | 728 | 71.8 | 249 | 76.6 | 476 | 69.5 | 570 | 71.1 | 148 | 74.4 | 214 | 80.1 | 78 | 83.0 | 146 | 63.2 | 57 | 60.6 | 103 | 71.5 | 119 | 69.2 |
|  | Yes | 286 | 28.2 | 76 | 23.4 | 209 | 30.5 | 232 | 28.9 | 51 | 25.6 | 53 | 19.9 | 16 | 17.0 | 85 | 36.8 | 37 | 39.4 | 41 | 28.5 | 53 | 30.8 |
| Parent drink problem | | | |  |  |  |  |  |  |  |  |  |  |  |  |  |  |  |  |  |  |  |  |
|  | No | 950 | 93.5 | 306 | 94.2 | 640 | 93.2 | 752 | 93.5 | 186 | 93.5 | 260 | 97.4 | 89 | 93.7 | 205 | 88.7 | 86 | 91.5 | 136 | 94.4 | 162 | 93.6 |
|  | Yes | 66 | 6.5 | 19 | 5.8 | 47 | 6.8 | 52 | 6.5 | 13 | 6.5 | 7 | 2.6 | 6 | 6.3 | 26 | 11.3 | 8 | 8.5 | 8 | 5.6 | 11 | 6.4 |
| **Housing** | |  |  |  |  |  |  |  |  |  |  |  |  |  |  |  |  |  |  |  |  |  |  |
| Housing problems | |  |  |  |  |  |  |  |  |  |  |  |  |  |  |  |  |  |  |  |  |  |  |
|  | 0 | 585 | 54.5 | 199 | 57.2 | 382 | 53.1 | 464 | 55.0 | 109 | 50.5 | 149 | 53.4 | 51 | 50.5 | 144 | 60.5 | 59 | 57.8 | 80 | 51.6 | 93 | 50.5 |
|  | 1 | 241 | 22.4 | 80 | 23.0 | 160 | 22.2 | 193 | 22.9 | 46 | 21.3 | 70 | 25.1 | 26 | 25.7 | 55 | 23.1 | 17 | 16.7 | 25 | 16.1 | 46 | 25.0 |
|  | 2 | 140 | 13.0 | 41 | 11.8 | 99 | 13.8 | 106 | 12.6 | 34 | 15.7 | 36 | 12.9 | 12 | 11.9 | 21 | 8.8 | 20 | 19.6 | 31 | 20.0 | 20 | 10.9 |
|  | 3+ | 108 | 10.1 | 28 | 8.0 | 79 | 11.0 | 80 | 9.5 | 27 | 12.5 | 24 | 8.6 | 12 | 11.9 | 18 | 7.6 | 6 | 5.9 | 19 | 12.3 | 25 | 13.6 |
| Easy internet access | |  |  |  |  |  |  |  |  |  |  |  |  |  |  |  |  |  |  |  |  |  |  |
|  | No | 10 | 0.9 | 5 | 1.4 | 5 | 0.7 | 4 | 0.5 | 6 | 2.8 | 2 | 0.7 | 4 | 4.0 | 1 | 0.4 | 1 | 1.0 | 0 | 0.0 | 2 | 1.1 |
|  | Yes | 1061 | 99.1 | 341 | 98.6 | 714 | 99.3 | 836 | 99.5 | 210 | 97.2 | 275 | 99.3 | 97 | 96.0 | 237 | 99.6 | 101 | 99.0 | 155 | 100 | 181 | 98.9 |
| **Relationships** | |  |  |  |  |  |  |  |  |  |  |  |  |  |  |  |  |  |  |  |  |  |  |
| Change in family relats. | | |  |  |  |  |  |  |  |  |  |  |  |  |  |  |  |  |  |  |  |  |  |
|  | Lot worse | 28 | 2.6 | 10 | 2.9 | 18 | 2.5 | 19 | 2.3 | 8 | 3.7 | 5 | 1.8 | 4 | 4.0 | 7 | 2.9 | 4 | 3.9 | 3 | 1.9 | 5 | 2.7 |
|  | Little worse | 135 | 12.6 | 37 | 10.7 | 98 | 13.6 | 106 | 12.6 | 28 | 13.0 | 28 | 10.1 | 11 | 10.9 | 41 | 17.2 | 10 | 9.8 | 21 | 13.6 | 23 | 12.6 |
|  | Same | 571 | 53.4 | 206 | 59.5 | 360 | 50.1 | 452 | 53.8 | 113 | 52.6 | 153 | 55.2 | 56 | 55.4 | 131 | 55.0 | 54 | 52.9 | 73 | 47.4 | 95 | 51.9 |
|  | Little better | 239 | 22.3 | 72 | 20.8 | 166 | 23.1 | 196 | 23.3 | 38 | 17.7 | 51 | 18.4 | 21 | 20.8 | 51 | 21.4 | 30 | 29.4 | 43 | 27.9 | 40 | 21.9 |
|  | Lot better | 97 | 9.1 | 21 | 6.1 | 76 | 10.6 | 67 | 8.0 | 28 | 13.0 | 40 | 14.4 | 9 | 8.9 | 8 | 3.4 | 4 | 3.9 | 14 | 9.1 | 20 | 10.9 |
| Freq. argue w. parents | | |  |  |  |  |  |  |  |  |  |  |  |  |  |  |  |  |  |  |  |  |  |
|  | Never | 138 | 13.5 | 56 | 16.9 | 80 | 11.6 | 99 | 12.2 | 34 | 17.1 | 45 | 16.9 | 15 | 15.6 | 16 | 6.9 | 15 | 15.8 | 16 | 11.0 | 29 | 16.3 |
|  | Hardly ever | 325 | 31.7 | 124 | 37.5 | 200 | 29.0 | 263 | 32.3 | 59 | 29.6 | 97 | 36.3 | 35 | 36.5 | 60 | 25.9 | 27 | 28.4 | 47 | 32.4 | 54 | 30.3 |
|  | < once a week | 285 | 27.8 | 86 | 26.0 | 199 | 28.8 | 236 | 29.0 | 48 | 24.1 | 62 | 23.2 | 27 | 28.1 | 73 | 31.5 | 24 | 25.3 | 40 | 27.6 | 57 | 32.0 |
|  | > once a week | 202 | 19.7 | 50 | 15.1 | 151 | 21.9 | 155 | 19.1 | 44 | 22.1 | 41 | 15.4 | 14 | 14.6 | 65 | 28.0 | 23 | 24.2 | 32 | 22.1 | 25 | 14.0 |
|  | Most days | 75 | 7.3 | 15 | 4.5 | 60 | 8.7 | 60 | 7.4 | 14 | 7.0 | 22 | 8.2 | 5 | 5.2 | 18 | 7.8 | 6 | 6.3 | 10 | 6.9 | 13 | 7.3 |
| Feel lonely | |  |  |  |  |  |  |  |  |  |  |  |  |  |  |  |  |  |  |  |  |  |  |
|  | Not at all | 414 | 38.7 | 179 | 51.6 | 233 | 32.5 | 328 | 39.0 | 80 | 37.0 | 130 | 46.8 | 34 | 33.7 | 87 | 36.6 | 41 | 40.2 | 48 | 31.2 | 67 | 36.6 |
|  | Slightly | 361 | 33.7 | 104 | 30.0 | 256 | 35.7 | 291 | 34.6 | 66 | 30.6 | 83 | 29.9 | 34 | 33.7 | 91 | 38.2 | 32 | 31.4 | 56 | 36.4 | 60 | 32.8 |
|  | Moderately | 209 | 19.5 | 47 | 13.5 | 160 | 22.3 | 158 | 18.8 | 47 | 21.8 | 47 | 16.9 | 21 | 20.8 | 45 | 18.9 | 20 | 19.6 | 35 | 22.7 | 39 | 21.3 |
|  | Very | 87 | 8.1 | 17 | 4.9 | 69 | 9.6 | 63 | 7.5 | 23 | 10.6 | 18 | 6.5 | 12 | 11.9 | 15 | 6.3 | 9 | 8.8 | 15 | 9.7 | 17 | 9.3 |
| **Routines** | |  |  |  |  |  |  |  |  |  |  |  |  |  |  |  |  |  |  |  |  |  |  |
| Stable routine | | | |  |  |  |  |  |  |  |  |  |  |  |  |  |  |  |  |  |  |  |  |
|  | No | 208 | 20.1 | 50 | 14.9 | 158 | 22.8 | 162 | 19.8 | 44 | 21.6 | 59 | 21.7 | 30 | 30.9 | 24 | 10.3 | 17 | 17.9 | 33 | 22.4 | 44 | 24.7 |
|  | Little | 322 | 31.1 | 92 | 27.4 | 229 | 33.0 | 255 | 31.2 | 65 | 31.9 | 87 | 32.0 | 28 | 28.9 | 75 | 32.2 | 27 | 28.4 | 41 | 27.9 | 62 | 34.8 |
|  | Moderate | 310 | 30.0 | 121 | 36.0 | 188 | 27.1 | 238 | 29.1 | 66 | 32.4 | 81 | 29.8 | 25 | 25.8 | 75 | 32.2 | 25 | 26.3 | 46 | 31.3 | 53 | 29.8 |
|  | Lot | 122 | 11.8 | 41 | 12.2 | 80 | 11.5 | 104 | 12.7 | 16 | 7.8 | 26 | 9.6 | 8 | 8.2 | 38 | 16.3 | 18 | 18.9 | 15 | 10.2 | 15 | 8.4 |
|  | Great deal | 72 | 7.0 | 32 | 9.5 | 39 | 5.6 | 58 | 7.1 | 13 | 6.4 | 19 | 7.0 | 6 | 6.2 | 21 | 9.0 | 8 | 8.4 | 12 | 8.2 | 4 | 2.2 |
| Difficulty sleeping | |  |  |  |  |  |  |  |  |  |  |  |  |  |  |  |  |  |  |  |  |  |  |
|  | No | 376 | 35.2 | 156 | 45.0 | 219 | 30.6 | 293 | 34.9 | 79 | 36.7 | 106 | 38.4 | 34 | 33.7 | 81 | 34.0 | 35 | 34.3 | 57 | 37.0 | 58 | 31.7 |
|  | A little | 263 | 24.6 | 89 | 25.6 | 173 | 24.2 | 221 | 26.3 | 38 | 17.7 | 61 | 22.1 | 21 | 20.8 | 65 | 27.3 | 29 | 28.4 | 31 | 20.1 | 51 | 27.9 |
|  | Sometimes | 189 | 17.7 | 49 | 14.1 | 138 | 19.3 | 139 | 16.6 | 46 | 21.4 | 51 | 18.5 | 26 | 25.7 | 38 | 16.0 | 16 | 15.7 | 25 | 16.2 | 31 | 16.9 |
|  | Quite often | 126 | 11.8 | 32 | 9.2 | 94 | 13.1 | 96 | 11.4 | 30 | 14.0 | 29 | 10.5 | 12 | 11.9 | 30 | 12.6 | 12 | 11.8 | 20 | 13.0 | 23 | 12.6 |
|  | A lot | 115 | 10.8 | 21 | 6.1 | 92 | 12.8 | 90 | 10.7 | 22 | 10.2 | 29 | 10.5 | 8 | 7.9 | 24 | 10.1 | 10 | 9.8 | 21 | 13.6 | 20 | 10.9 |
| Change in exercise | |  |  |  |  |  |  |  |  |  |  |  |  |  |  |  |  |  |  |  |  |  |  |
|  | Much less | 212 | 20.1 | 64 | 18.7 | 147 | 20.8 | 175 | 21.0 | 35 | 16.7 | 63 | 23.1 | 22 | 22.2 | 34 | 14.3 | 22 | 22.2 | 39 | 25.7 | 30 | 16.6 |
|  | A bit less | 197 | 18.7 | 48 | 14.0 | 148 | 20.9 | 156 | 18.8 | 36 | 17.1 | 49 | 17.9 | 10 | 10.1 | 56 | 23.5 | 25 | 25.3 | 23 | 15.1 | 29 | 16.0 |
|  | The same | 263 | 24.9 | 103 | 30.0 | 160 | 22.6 | 198 | 23.8 | 63 | 30.0 | 61 | 22.3 | 33 | 33.3 | 57 | 23.9 | 21 | 21.2 | 39 | 25.7 | 50 | 27.6 |
|  | A bit more | 221 | 20.9 | 78 | 22.7 | 141 | 19.9 | 173 | 20.8 | 45 | 21.4 | 51 | 18.7 | 17 | 17.2 | 60 | 25.2 | 21 | 21.2 | 30 | 19.7 | 38 | 21.0 |
|  | Much more | 163 | 15.4 | 50 | 14.6 | 112 | 15.8 | 130 | 15.6 | 31 | 14.8 | 49 | 17.9 | 17 | 17.2 | 31 | 13.0 | 10 | 10.1 | 21 | 13.8 | 34 | 18.8 |

FSM, Free School Meals.

**Table S4.** Reported worries or concerns (note: frequencies and percentages are descriptive, not weighted).

|  | **Total** | | **Sex** | | | | **Free School Meals** | | | | **Ethnic Group** | | | | | | | | | | | |
| --- | --- | --- | --- | --- | --- | --- | --- | --- | --- | --- | --- | --- | --- | --- | --- | --- | --- | --- | --- | --- | --- | --- |
|  | **(n, 1074)** | | **Boys  (n, 348)** | | **Girls (n, 720)** | | **No (n, 843)** | | **Yes (n, 216)** | | **B. African (n, 279)** | | **B. Caribbean (n, 101)** | | **Br. White (n, 238)** | | **non-Br. W. (n, 102)** | | **Mixed (n, 155)** | | **Other (n, 184)** | |
|  | **n** | **%** | **n** | **%** | **n** | **%** | **n** | **%** | **n** | **%** | **n** | **%** | **n** | **%** | **n** | **%** | **n** | **%** | **n** | **%** | **n** | **%** |
| Having to stay home | 215 | 20.6 | 48 | 14.2 | 164 | 23.4 | 161 | 19.6 | 50 | 24.3 | 60 | 22.2 | 20 | 20.6 | 44 | 18.6 | 22 | 22.3 | 30 | 20.2 | 35 | 19.8 |
| Not seeing friends | 345 | 33.2 | 72 | 21.4 | 271 | 38.7 | 269 | 32.7 | 70 | 33.9 | 83 | 30.6 | 35 | 36.1 | 79 | 33.4 | 33 | 33.6 | 51 | 34.4 | 60 | 33.5 |
| Family might get sick | 404 | 39 | 96 | 28.5 | 307 | 44.1 | 306 | 37.2 | 96 | 47.5 | 111 | 41.1 | 36 | 37.5 | 79 | 33.2 | 32 | 32.3 | 64 | 43.5 | 80 | 45.7 |
| Falling behind with schoolwork | 397 | 38.3 | 79 | 23.7 | 317 | 45.3 | 308 | 37.5 | 85 | 41.7 | 117 | 43.3 | 34 | 35.4 | 75 | 31.6 | 29 | 29.3 | 49 | 33.3 | 90 | 50.8 |
| Spending more time with family | 141 | 13.5 | 39 | 11.5 | 101 | 14.4 | 102 | 12.4 | 36 | 17.3 | 36 | 13.3 | 14 | 14.8 | 21 | 8.9 | 14 | 14.2 | 27 | 18 | 26 | 14.6 |
| People might die if they get sick | 378 | 36.4 | 90 | 26.8 | 285 | 40.9 | 292 | 35.6 | 83 | 40.4 | 105 | 38.9 | 39 | 40.7 | 74 | 31.2 | 31 | 31.3 | 64 | 43.5 | 61 | 34.7 |
| Parent job loss | 182 | 17.6 | 46 | 13.8 | 134 | 19.2 | 133 | 16.2 | 47 | 24 | 56 | 21 | 14 | 14.6 | 26 | 11.1 | 13 | 13.2 | 31 | 21.3 | 39 | 22.2 |
| Having enough to eat | 89 | 8.6 | 27 | 8.1 | 62 | 8.8 | 63 | 7.6 | 25 | 12.3 | 27 | 10 | 13 | 13.6 | 15 | 6.4 | 4 | 4.0 | 13 | 8.9 | 17 | 9.6 |
| Conflict between parents | 90 | 8.8 | 18 | 5.4 | 72 | 10.3 | 72 | 8.8 | 17 | 8.5 | 20 | 7.4 | 4 | 4.3 | 25 | 10.7 | 10 | 10.1 | 16 | 10.8 | 15 | 8.5 |
| Conflict with parents | 123 | 11.9 | 18 | 5.4 | 104 | 14.9 | 97 | 11.8 | 24 | 12 | 35 | 13 | 8 | 8.4 | 24 | 10.2 | 11 | 11.2 | 24 | 16.2 | 20 | 11.4 |
| Sibling conflicts | 130 | 12.7 | 21 | 6.3 | 109 | 15.9 | 96 | 11.9 | 33 | 16.5 | 37 | 13.9 | 8 | 8.4 | 23 | 9.9 | 6 | 6.3 | 26 | 17.9 | 29 | 16.8 |
| Getting into college/university | 293 | 28.9 | 68 | 20.6 | 224 | 32.9 | 220 | 27.3 | 71 | 36.3 | 97 | 37 | 29 | 31.2 | 37 | 15.7 | 27 | 27.6 | 39 | 26.9 | 62 | 36.4 |
| Grades/exams | 520 | 50.2 | 114 | 34.0 | 404 | 58.0 | 396 | 48.3 | 117 | 57.6 | 153 | 57.1 | 55 | 57.3 | 82 | 34.8 | 45 | 45.4 | 74 | 50 | 106 | 59.9 |
| Being evicted | 21 | 2.1 | 10 | 3 | 11 | 1.6 | 12 | 1.5 | 9 | 4.5 | 5 | 1.9 | 3 | 3.1 | 1 | 0.4 | 2 | 2 | 4 | 2.8 | 6 | 3.4 |
| Gaining weight | 285 | 27.4 | 47 | 13.9 | 236 | 33.7 | 215 | 26.1 | 65 | 31.7 | 66 | 24.4 | 27 | 27.9 | 59 | 24.9 | 25 | 25.2 | 49 | 32.9 | 55 | 31.3 |
| Getting enough exercise | 259 | 24.8 | 61 | 18.1 | 196 | 27.9 | 199 | 24.2 | 57 | 27.6 | 64 | 23.6 | 26 | 26.8 | 50 | 21.1 | 25 | 25.3 | 47 | 31.3 | 43 | 24.3 |
| Mental health | 260 | 25.2 | 45 | 13.5 | 213 | 30.4 | 206 | 25 | 51 | 24.9 | 57 | 21.2 | 21 | 21.6 | 57 | 24.1 | 26 | 26.2 | 51 | 34.3 | 45 | 25.5 |
| Having enough money | 170 | 16.3 | 46 | 13.8 | 123 | 17.5 | 117 | 14.2 | 51 | 25 | 58 | 21.4 | 24 | 24.7 | 22 | 9.4 | 7 | 7.0 | 30 | 20.3 | 27 | 15.3 |
| Losing internet access | 118 | 11.3 | 46 | 13.8 | 71 | 10.1 | 88 | 10.7 | 26 | 12.8 | 30 | 11.1 | 12 | 12.4 | 23 | 9.7 | 12 | 12.1 | 17 | 11.5 | 20 | 11.3 |
| Getting medication | 40 | 3.9 | 14 | 4.2 | 26 | 3.7 | 32 | 3.9 | 8 | 4.0 | 13 | 4.9 | 8 | 8.3 | 3 | 1.3 | 2 | 2.0 | 9 | 6.2 | 5 | 2.9 |
| Future plans | 328 | 31.8 | 64 | 19.2 | 262 | 37.6 | 248 | 30.2 | 75 | 37.3 | 101 | 37.5 | 33 | 34.4 | 51 | 21.7 | 20 | 20.2 | 55 | 37.4 | 63 | 36.2 |
| Missing key events (e.g., graduation) | 303 | 29.4 | 69 | 20.7 | 233 | 33.5 | 239 | 29.2 | 59 | 29.2 | 78 | 29 | 26 | 27.1 | 71 | 30.4 | 29 | 29.3 | 39 | 26.5 | 56 | 32 |
| Romantic relationships | 101 | 9.9 | 35 | 10.7 | 66 | 9.6 | 77 | 9.6 | 24 | 12.3 | 22 | 8.3 | 10 | 10.6 | 23 | 10.1 | 9 | 9.2 | 19 | 13.1 | 17 | 10 |
| Young people get unfair reputation | 117 | 11.4 | 42 | 12.6 | 74 | 10.7 | 91 | 11.2 | 25 | 12.3 | 28 | 10.5 | 13 | 13.7 | 27 | 11.6 | 10 | 10.1 | 16 | 10.9 | 22 | 12.5 |

Frequencies and percentages are descriptive (not weighted).

Responses are to the question: "In the past 4 weeks, due to the COVID-19 pandemic, to what extent have you been concerned about the following: … " This table presents the frequency and proportion that responded a lot or a great deal.

**Table S5.** Reported positives (note: frequencies and percentages are descriptive, not weighted).

|  | **Total** | | **Sex** | | | | **Free School Meals** | | | | **Ethnic Group** | | | | | | | | | | | |
| --- | --- | --- | --- | --- | --- | --- | --- | --- | --- | --- | --- | --- | --- | --- | --- | --- | --- | --- | --- | --- | --- | --- |
|  | **(n, 1074)** | | **Boys  (n, 348)** | | **Girls (n, 720)** | | **No (n, 843)** | | **Yes (n, 216)** | | **B. African (n, 279)** | | **B. Caribbean (n, 101)** | | **Br. White (n, 238)** | | **non-Br. W. (n, 102)** | | **Mixed (n, 155)** | | **Other (n, 184)** | |
|  | **n** | **%** | **n** | **%** | **n** | **%** | **n** | **%** | **n** | **%** | **n** | **%** | **n** | **%** | **n** | **%** | **n** | **%** | **n** | **%** | **n** | **%** |
| Feel less exam stress | 662 | 69.3 | 193 | 62.2 | 465 | 72.5 | 504 | 67.3 | 145 | 75.1 | 184 | 73.6 | 63 | 71.6 | 138 | 64.5 | 60 | 66.6 | 93 | 67.9 | 117 | 70.9 |
| Feel less social pressure | 501 | 52.0 | 148 | 46.5 | 349 | 54.3 | 400 | 52.3 | 94 | 50.0 | 118 | 47.6 | 45 | 51.1 | 128 | 56.9 | 49 | 53.9 | 77 | 54.6 | 77 | 48.2 |
| Enjoy more time to self | 916 | 89.8 | 283 | 86.3 | 629 | 91.4 | 727 | 90.1 | 178 | 88.6 | 238 | 88.1 | 83 | 88.3 | 210 | 90.1 | 86 | 91.5 | 135 | 92.5 | 154 | 90.0 |
| Enjoy time to learn new skills | 805 | 80.3 | 252 | 78.1 | 549 | 81.4 | 632 | 79.7 | 163 | 82.7 | 217 | 82.2 | 78 | 83.0 | 171 | 74.7 | 79 | 85.0 | 111 | 77.7 | 140 | 83.3 |
| Enjoy more time with family | 855 | 83.8 | 275 | 83.6 | 577 | 84.0 | 672 | 83.4 | 172 | 85.5 | 220 | 82.4 | 78 | 82.1 | 193 | 83.2 | 76 | 80.9 | 126 | 86.9 | 154 | 88.0 |
| Enjoy flexi online study | 660 | 67.0 | 215 | 67.2 | 443 | 67.1 | 526 | 67.7 | 125 | 64.1 | 175 | 68.4 | 60 | 66.7 | 162 | 71.4 | 62 | 68.9 | 91 | 64.6 | 102 | 60.7 |
| Less anxious in lessons | 534 | 57.1 | 157 | 52.3 | 374 | 59.2 | 423 | 57.0 | 101 | 55.8 | 127 | 52.2 | 49 | 58.4 | 122 | 56.5 | 56 | 63.7 | 82 | 60.3 | 93 | 58.5 |
| Reconnect with old friends | 605 | 67.0 | 211 | 72.0 | 391 | 64.6 | 477 | 66.9 | 117 | 66.1 | 168 | 69.4 | 61 | 74.4 | 132 | 64.7 | 54 | 65.1 | 82 | 64.6 | 99 | 65.2 |
| Easier to connect w. other time zones | 577 | 65.2 | 197 | 68.2 | 376 | 63.6 | 445 | 63.4 | 121 | 70.3 | 158 | 66.6 | 52 | 67.6 | 110 | 57.0 | 57 | 67.1 | 83 | 62.8 | 108 | 72.0 |
| Better sense of community in neighbourhood | 545 | 57.1 | 186 | 59.6 | 356 | 55.7 | 432 | 57.2 | 103 | 55.1 | 133 | 52.5 | 46 | 54.1 | 144 | 65.8 | 54 | 60.6 | 74 | 53.6 | 88 | 55.0 |
| Prefer virtual school | 498 | 51.2 | 165 | 52.4 | 330 | 50.3 | 392 | 50.7 | 98 | 52.2 | 124 | 49.2 | 43 | 49.4 | 121 | 53.3 | 53 | 58.3 | 79 | 56.4 | 71 | 43.0 |
| Realise what's important in life | 845 | 83.9 | 247 | 76.7 | 594 | 87.2 | 672 | 84.0 | 164 | 83.7 | 230 | 87.1 | 79 | 85.9 | 183 | 79.9 | 79 | 83.1 | 117 | 80.7 | 148 | 87.0 |
| Enjoy being away from certain people at school | 799 | 79.4 | 238 | 73.0 | 557 | 82.4 | 628 | 78.9 | 162 | 81.9 | 211 | 79.3 | 82 | 90.1 | 186 | 81.2 | 73 | 78.5 | 120 | 83.4 | 118 | 68.6 |
| Enjoy doing more physical exercise | 619 | 65.7 | 213 | 69.6 | 403 | 63.8 | 485 | 65.2 | 124 | 66.7 | 159 | 64.9 | 59 | 70.2 | 150 | 69.1 | 58 | 68.2 | 83 | 59.3 | 102 | 64.2 |

Frequencies and percentages are descriptive (not weighted).

Responses to the question: "Some people have found that the social distancing measures have had some positive effects on their daily life. To what extent have you found the following to be true: …". This table presents the frequency and proportion that responded a somewhat true or certainly true.

**Table S6.** Weighted prevalence estimates and 95% confidence intervals of depression, anxiety, and lifetime self-harm pre- and mid-covid-19.

|  | | **Depression (SMFQ)** | | | | **Anxiety (GAD-7)** | | | | **Self-harm (lifetime)** | | | |
| --- | --- | --- | --- | --- | --- | --- | --- | --- | --- | --- | --- | --- | --- |
|  | | **T3**  (2018-19) | | **T4 (Covid-19)**  (2020) | | **T3**  (2018-19) | | **T4 (Covid-19)**  (2020) | | **T3**  (2018-19) | | **T4 (Covid-19)**  (2020) | |
|  | | n* | %** (95% CI) | n* | %** (95% CI) | n* | %** (95% CI) | n* | %** (95% CI) | n* | %** (95% CI) | n* | %** (95% CI) |
| **Overall** | | 232 | 27.8 (22.6, 33.7) | 242 | 22.6 (19.3, 26.4) | 173 | 20.5 (17.3, 24.3) | 185 | 17.3 (14.0, 21.0) | 142 | 17.0 (12.7, 22.4) | 150 | 15.1 (11.2, 20.1) |
|  | |  |  |  |  |  |  |  |  |  |  |  |  |
| Sex | |  |  |  |  |  |  |  |  |  |  |  |  |
|  | Boys | 63 | 16.3 (10.8, 23.9) | 67 | 13.8 (11.0, 17.2) | 39 | 10.2 (7.0, 14.8) | 50 | 10.2 (6.9, 14.8) | 41 | 10.5 (6.3, 16.9) | 42 | 9.2 (5.9, 14.1) |
|  | Girls | 169 | 37.6 (32.5, 42.9) | 175 | 30.0 (24.6, 35.9) | 134 | 29.0 (24.9, 33.6) | 135 | 23.1 (20.3, 26.2) | 101 | 22.7 (16.0, 31.1) | 108 | 20.1 (14.5, 27.1) |
|  | |  |  |  |  |  |  |  |  |  |  |  |  |
| Free school meals | |  |  |  |  |  |  |  |  |  |  |  |  |
|  | No | 166 | 26.5 (21.2, 32.7) | 176 | 22.7 (18.4, 27.6) | 125 | 20.0 (16.6, 23.8) | 126 | 16.2 (13.3, 19.7) | 96 | 15.7 (10.7, 22.5) | 105 | 14.6 (10.4, 20.2) |
|  | Yes | 66 | 31.6 (20.2, 45.7) | 66 | 22.4 (16.0, 30.6) | 48 | 22.3 (15.9, 30.2) | 59 | 20.0 (12.4, 30.7) | 46 | 20.4 (14.5, 28.0) | 45 | 16.3 (9.4, 26.8) |
|  | |  |  |  |  |  |  |  |  |  |  |  |  |
| Ethnic group | |  |  |  |  |  |  |  |  |  |  |  |  |
|  | Black African | 45 | 20.1 (14.1, 27.8) | 51 | 18.4 (13.3, 25.1) | 34 | 15.0 (10.6, 20.8) | 42 | 15.2 (10.6, 21.2) | 29 | 9.3 (5.9, 14.2) | 20 | 7.8 (5.0, 11.9) |
|  | Black Caribbean | 29 | 28.6 (14.7, 48.2) | 35 | 24.0 (14.7, 36.7) | 27 | 26.1 (16.8, 38.2) | 27 | 18.2 (8.1, 36.1) | 23 | 22.1 (12.3, 36.3) | 33 | 23.5 (13.7, 37.5) |
|  | British White | 38 | 24.9 (19.7, 30.9) | 43 | 24.4 (19.9, 29.4) | 27 | 18.3 (11.6, 27.8) | 37 | 20.6 (13.1, 30.9) | 22 | 20.4 (16.5, 25.1) | 35 | 20.1 (14.1, 27.8) |
|  | Non-British White | 28 | 33.8 (25.2, 43.6) | 27 | 24.2 (18.0, 31.6) | 20 | 23.4 (18.7, 29.0) | 18 | 16.0 (9.8, 25.0) | 16 | 19.3 (10.1, 33.8) | 19 | 17.4 (7.8, 34.3) |
|  | Mixed | 47 | 37.6 (28.6, 47.7) | 41 | 24.5 (14.7, 38.0) | 30 | 22.6 (18.2, 27.7) | 28 | 17.1 (10.5, 26.5) | 29 | 22.1 (15.5, 30.4) | 28 | 19.2 (11.7, 29.8) |
|  | Other | 45 | 30.3 (27.3, 33.4) | 45 | 23.5 (16.8, 32.0) | 35 | 23.8 (16.5, 32.9) | 33 | 17.3 (11.8, 24.8) | 23 | 16.3 (9.3, 27.2) | 16 | 9.3 (6.3, 13.5) |
|  | |  |  |  |  |  |  |  |  |  |  |  |  |
| School year at T4 | |  |  |  |  |  |  |  |  |  |  |  |  |
|  | Year 8-9 | 40 | 32.0 (18.4, 49.5) | 31 | 20.3 (18.8, 21.7) | 25 | 20.6 (13.9, 29.3) | 21 | 13.5 (9.4, 18.9) | 25 | 21.6 (18.8, 24.7) | 25 | 17.4 (14.6, 20.7) |
|  | Year 10 | 61 | 29.4 (26.2, 32.9) | 54 | 22.5 (16.5, 30.1) | 43 | 20.9 (16.9, 25.4) | 41 | 17.3 (10.7, 26.6) | 39 | 20.3 (14.6, 27.5) | 38 | 17.6 (10.1, 28.8) |
|  | Year 11 | 75 | 26.9 (19.5, 35.8) | 86 | 24.2 (20.9, 27.8) | 59 | 20.8 (15.0, 28.2) | 62 | 17.3 (13.0, 22.7) | 44 | 14.7 (9.9, 21.4) | 44 | 13.1 (9.4, 18.1) |
|  | Year 12-13 | 48 | 24.6 (16.3, 35.4) | 58 | 20.4 (13.6, 29.4) | 39 | 19.8 (15.2, 25.3) | 52 | 18.2 (13.6, 23.9) | 29 | 14.1 (9.9, 19.7) | 39 | 14.4 (10.0, 20.4) |

All percentages are weighted, using inverse probability weights

Robust standard errors are used to account for clustering of pupils within schools

T, Time

* Number calibrated to weights and rounded to nearest whole number

** Weighted %

**Figure S1.** Weighted prevalence estimates and 95% confidence intervals of depression, anxiety, and lifetime self-harm at each time point.

(a) Depression

(b) Anxiety

(c) Self-harm

Note: GAD-7 (anxiety) and SMFQ (depression) only administered to a subset of schools at T2, so data are presented for T1, T3, T4 (available for T2 on request).

**Table S7.** Social circumstances and experiences pre-covid-19 (note: frequencies and percentages are descriptive, not weighted).

|  | | **Total** | | | | | | **Sex** | | | | | | | | | | | |
| --- | --- | --- | --- | --- | --- | --- | --- | --- | --- | --- | --- | --- | --- | --- | --- | --- | --- | --- | --- |
|  |  |  |  |  |  |  |  | **Boys** | | | | | | **Girls** | | | | | |
|  | | **T1** | | **T2** | | **T3** | | **T1** | | **T2** | | **T3** | | **T1** | | **T2** | | **T3** | |
|  |  | **n** | **%** | **n** | **%** | **n** | **%** | **n** | **%** | **n** | **%** | **n** | **%** | **n** | **%** | **n** | **%** | **n** | **%** |
| Family Affluence | |  |  |  |  |  |  |  |  |  |  |  |  |  |  |  |  |  |  |
|  | High | 599 | 61.6 | 536 | 59.4 | 504 | 56.7 | 205 | 63.3 | 189 | 63.2 | 170 | 61.4 | 394 | 60.7 | 347 | 57.5 | 334 | 54.6 |
|  | Low, moderate | 374 | 38.4 | 367 | 40.6 | 385 | 43.3 | 119 | 36.7 | 110 | 36.8 | 107 | 38.6 | 255 | 39.3 | 257 | 42.6 | 278 | 45.4 |
| Household financial problems | |  |  |  |  |  |  |  |  |  |  |  |  |  |  |  |  |  |  |
|  | No | 713 | 87.0 | 778 | 88.4 | 735 | 89.0 | 223 | 88.1 | 249 | 90.2 | 227 | 89.7 | 490 | 86.4 | 529 | 87.6 | 508 | 88.7 |
|  | Yes | 107 | 13.0 | 102 | 11.6 | 91 | 11.0 | 30 | 11.9 | 27 | 9.8 | 26 | 10.3 | 77 | 13.6 | 75 | 12.4 | 65 | 11.3 |
| Parents often argue | |  |  |  |  |  |  |  |  |  |  |  |  |  |  |  |  |  |  |
|  | No | 500 | 62.5 | 619 | 70.8 | 618 | 75.1 | 164 | 66.1 | 205 | 75.4 | 204 | 81.3 | 336 | 60.9 | 414 | 68.8 | 414 | 72.4 |
|  | Yes | 300 | 37.5 | 255 | 29.2 | 205 | 24.9 | 84 | 33.9 | 67 | 24.6 | 47 | 18.7 | 216 | 39.1 | 188 | 31.2 | 158 | 27.6 |
| Parent drinking problems | |  |  |  |  |  |  |  |  |  |  |  |  |  |  |  |  |  |  |
|  | No | 777 | 94.0 | 838 | 94.7 | 787 | 95.0 | 241 | 94.1 | 268 | 97.1 | 242 | 95.3 | 536 | 93.9 | 570 | 93.6 | 545 | 94.9 |
|  | Yes | 50 | 6.0 | 47 | 5.3 | 41 | 5.0 | 15 | 5.9 | 8 | 2.9 | 12 | 4.7 | 35 | 6.1 | 39 | 6.4 | 29 | 5.1 |
| Victim of bullying | |  |  |  |  |  |  |  |  |  |  |  |  |  |  |  |  |  |  |
|  | No | 741 | 77.8 | 845 | 83.8 | 811 | 85.5 | 240 | 77.2 | 259 | 79.7 | 242 | 83.5 | 501 | 78.2 | 580 | 85.7 | 564 | 86.2 |
|  | Yes | 211 | 22.2 | 163 | 16.2 | 138 | 14.5 | 71 | 22.8 | 66 | 20.3 | 48 | 16.6 | 140 | 21.8 | 97 | 14.3 | 90 | 13.8 |
| Lonely | |  |  |  |  |  |  |  |  |  |  |  |  |  |  |  |  |  |  |
|  | No | 739 | 83.1 | 661 | 78.8 | 559 | 73.4 | 242 | 83.7 | 213 | 84.5 | 177 | 81.2 | 497 | 82.8 | 448 | 76.3 | 382 | 70.2 |
|  | Yes | 150 | 16.9 | 178 | 21.2 | 203 | 26.6 | 47 | 16.3 | 39 | 15.5 | 41 | 18.8 | 103 | 17.2 | 139 | 23.7 | 162 | 29.8 |
| Mental health problems | |  |  |  |  |  |  |  |  |  |  |  |  |  |  |  |  |  |  |
|  | No | 768 | 79.5 | 765 | 80.8 | 778 | 81.0 | 263 | 81.7 | 250 | 82.0 | 265 | 83.3 | 505 | 78.4 | 515 | 80.2 | 512 | 79.9 |
|  | Yes | 198 | 20.5 | 182 | 19.2 | 182 | 19.0 | 59 | 18.3 | 55 | 18.0 | 53 | 16.7 | 139 | 21.6 | 127 | 19.8 | 129 | 20.1 |

T1, Time 1. T2, Time 2. T3, Time 3.

**Table S8.** Fixed effects regression models: within-person change pre-covid to mid-covid, overall, and by demographic group and select pre-Covid-19 risks, adjusted for age and passage of time (number of days between timepoints).

|  | | **SDQ total difficulties score** | | | **SDQ internalising score** | | | **SDQ externalising score** | | |
| --- | --- | --- | --- | --- | --- | --- | --- | --- | --- | --- |
|  | | **b** | **95% CI** | **p (interaction)** | **b** | **95% CI** | **p (interaction)** | **b** | **95% CI** | **p (interaction)** |
| **Overall** | | -0.06 | (-0.66, 0.54) |  | 0.04 | (-0.31, 0.39) |  | -0.27 | (-0.63, 0.10) |  |
| **Demographic** | |  |  |  |  |  |  |  |  |  |
| Sex | |  |  |  |  |  |  |  |  |  |
|  | Boys | -0.59 | (-1.37, 0.19) | **0.007** | -0.13 | (-0.57, 0.31) | **0.131** | -0.61 | (-1.07, -0.16) | **0.004** |
|  | Girls | 0.42 | (-0.19, 1.03) |  | 0.20 | (-0.18, 0.58) |  | 0.04 | (-0.35, 0.43) |  |
| Free school meals | |  |  |  |  |  |  |  |  |  |
|  | No | 0.02 | (-0.57, 0.62) | 0.394 | 0.03 | (-0.32, 0.38) | 0.851 | -0.16 | (-0.53, 0.21) | **0.069** |
|  | Yes | -0.36 | (-1.33, 0.61) |  | 0.08 | (-0.50, 0.66) |  | -0.65 | (-1.22, -0.07) |  |
| Ethnic group | |  |  |  |  |  |  |  |  |  |
|  | British White | 0.28 | (-0.55, 0.82) | 0.564 | -0.05 | (-0.48, 0.37) | 0.509 | 0.19 | (-0.27, 0.65) | **0.006** |
|  | Black African | -0.47 | (-1.34, 0.40) |  | -0.09 | (-0.60, 0.42) |  | -0.51 | (-1.02, 0.11) |  |
|  | Black Caribbean | -0.43 | (-1.90, 1.04) |  | 0.63 | (-0.20, 1.47) |  | -1.27 | (-2.12, -0.42) |  |
|  | non-British White | 0.45 | (-0.75, 1.65) |  | 0.16 | (-0.52, 0.84) |  | 0.14 | (-0.54, 0.83) |  |
|  | Mixed | 0.01 | (-0.89, 0.92) |  | 0.22 | (-0.33, 0.77) |  | -0.48 | (-1.04, 0.08) |  |
|  | Other | -0.14 | (-1.09, 0.80) |  | -0.11 | (-0.69, 0.46) |  | -0.19 | (-0.76, 0.38) |  |
| School year at T4 | |  |  |  |  |  |  |  |  |  |
|  | Year 8/9 | -0.35 | (-1.38, 0.68) | 0.091 | -0.13 | (-0.69, 0.43) | 0.636 | -0.45 | (-1.06, 0.17) | **0.023** |
|  | Year 10 | 0.39 | (-0.46, 1.24) |  | 0.20 | (-0.29, 0.68) |  | 0.06 | (-0.46, 0.59) |  |
|  | Year 11 | -0.50 | (-1.38, 0.38) |  | -0.04 | (-0.66, 0.59) |  | -0.61 | (-1.10, -0.13) |  |
|  | Year 12/13, post 16 | 0.95 | (-0.55, 2.44) |  | 0.38 | (-0.61, 1.37) |  | 0.33 | (-0.50, 1.16) |  |
| **Pre-Covid-19 risks** | |  |  |  |  |  |  |  |  |  |
| Family Affluence | |  |  |  |  |  |  |  |  |  |
|  | High | -0.22 | (-0.91, 0.48) | **0.016** | 0.07 | (-0.47, 0.33) | 0.211 | -0.34 | (-0.76, 0.07) | **0.012** |
|  | Low, moderate | -1.12 | (-1.89, -0.36) |  | -0.36 | (-0.82, 0.11) |  | -0.90 | (-1.37, -0.43) |  |
| Household financial problems | |  |  |  |  |  |  |  |  |  |
|  | No | 0.08 | (-0.60, 0.75) | 0.506 | 0.15 | (-0.26, 0.56) | 0.373 | -0.24 | (-0.65, 0.16) | 0.796 |
|  | Yes | -0.26 | (-1.35, 0.83) |  | -0.12 | (-0.73, 0.50) |  | -0.34 | (-1.09, 0.42) |  |
| Parents often argue | |  |  |  |  |  |  |  |  |  |
|  | No | 0.08 | (-0.63, 0.79) | 0.841 | 0.19 | (-0.24, 0.62) | 0.786 | -0.31 | (-0.73, 0.12) | 0.364 |
|  | Yes | 0.16 | (-0.68, 1.01) |  | 0.12 | (-0.38, 0.62) |  | -0.08 | (-0.63, 0.47) |  |
| Parent drinking problems | |  |  |  |  |  |  |  |  |  |
|  | No | 0.28 | (-0.64, 0.69) | 0.146 | 0.14 | (-0.27, 0.53) | 0.296 | -0.28 | (-0.69, 0.12) | 0.221 |
|  | Yes | 1.26 | (-0.48, 3.00) |  | 0.65 | (-0.33, 1.63) |  | 0.47 | (-0.74, 1.68) |  |
| Victim of bullying | |  |  |  |  |  |  |  |  |  |
|  | No | -0.07 | (-0.74, 0.60) | 0.561 | 0.09 | (-0.32, 0.49) | 0.581 | -0.33 | (-0.73, 0.07) | 0.694 |
|  | Yes | 0.25 | (-0.87, 1.37) |  | 0.27 | (-0.40, 0.94) |  | -0.21 | (-0.87, 0.45) |  |
| Lonely | |  |  |  |  |  |  |  |  |  |
|  | No | 0.26 | (-0.52, 1.03) | 0.076 | 0.24 | (-0.22, 0.70) | 0.032 | -0.18 | (-0.62, 0.26) | 0.500 |
|  | Yes | -0.54 | (-1.39, 0.31) |  | -0.31 | (-0.82, 0.20) |  | -0.37 | (-0.94, 0.19) |  |
| Mental health problems* (at T3) | |  |  |  |  |  |  |  |  |  |
|  | No | 0.24 | (-0.39, 0.87) | **0.002** | 0.22 | (-0.15, 0.59) | 0.008 | -0.11 | (-0.49, 0.27) | **0.015** |
|  | Yes | -1.04 | (-1.88, 0.20) |  | -0.46 | (-0.98, 0.06) |  | -0.77 | (-1.32, -0.22) |  |

All estimates are weighted, using inverse probability weights, and robust standard errors are used throughout. Model adjusted for age and days passed since data collection (entered as a linear and quadratic term). * Mental health problems defined as a score of 18 or more on the SDQ. b, regression coefficient.

**Table S9.** Fixed effects regression models: within-person change pre-covid to mid-covid, by mid-Covid-19 circumstances, experiences, and routines, adjusted for age and passage of time (number of days between timepoints)

|  | | **SDQ total difficulties score** | | | **SDQ internalising score** | | | **SDQ externalising score** | | |
| --- | --- | --- | --- | --- | --- | --- | --- | --- | --- | --- |
|  | | **b** | **95% CI** | **p (interaction)** | **b** | **95% CI** | **p (interaction)** | **b** | **95% CI** | **p (interaction)** |
| **Covid-19 infection** | |  |  |  |  |  |  |  |  |  |
| Self | |  |  |  |  |  |  |  |  |  |
|  | No | -0.14 | (-0.75, 0.47) | 0.289 | 0.02 | (-0.35, 0.39) | 0.473 | -0.33 | (-0.71, 0.05) | 0.186 |
|  | Yes | 0.63 | (-0.86, 2.13) |  | 0.31 | (-0.52, 1.14) |  | 0.18 | (-0.64, 1.01) |  |
| Family at home | |  |  |  |  |  |  |  |  |  |
|  | No | -0.16 | (-0.76, 0.44) | 0.483 | 0.04 | (-0.32, 0.39) | 0.938 | -0.38 | (-0.75, -0.01) | 0.131 |
|  | Yes | 0.19 | (-0.86, 1.23) |  | 0.01 | (-0.59, 0.62) |  | 0.04 | (-0.54, 0.63) |  |
| Other family | |  |  |  |  |  |  |  |  |  |
|  | No | -0.49 | (-1.10, 0.13) | **0.002** | -0.12 | (-0.49, 0.25) | 0.046 | -0.53 | (-0.92, -0.15) | **0.001** |
|  | Yes | 0.81 | (-0.05, 1.68) |  | 0.37 | (-0.13, 0.87) |  | 0.28 | (-0.22, 0.79) |  |
| **Household** | |  |  |  |  |  |  |  |  |  |
| Changes in income (number) | |  |  |  |  |  |  |  |  |  |
|  | 0 | -0.19 | (-0.81, 0.44) | 0.574 | -0.01 | (-0.38, 0.36) | 0.676 | -0.32 | (-0.70, 0.07) | 0.744 |
|  | 1 | 0.03 | (-0.91, 0.97) |  | 0.05 | (-0.48, 0.59) |  | -0.26 | (-0.80, 0.29) |  |
|  | 2+ | 0.44 | (-0.80, 1.68) |  | 0.31 | (-0.40, 1.02) |  | -0.03 | (-0.80, 0.73) |  |
| Financial problems | |  |  |  |  |  |  |  |  |  |
|  | No | -0.36 | (-0.96, 0.24) | **0.008** | -0.10 | (-0.45, 0.25) | **0.008** | -0.43 | (-0.81, -0.06) | **0.030** |
|  | Yes | 1.27 | (-0.04, 2.58) |  | 0.82 | (0.09, 1.55) |  | 0.31 | (-0.44, 1.10) |  |
| Parents often argue | |  |  |  |  |  |  |  |  |  |
|  | No | -0.49 | (-1.12, 0.14) | **0.005** | -0.12 | (-0.50, 0.26) | 0.054 | -0.57 | (-0.96, -0.18) | **< 0.001** |
|  | Yes | 0.74 | (-0.18, 1.66) |  | 0.38 | (-0.14, 0.90) |  | 0.25 | (-0.29, 0.78) |  |
| Parent drinking problems | |  |  |  |  |  |  |  |  |  |
|  | No | -0.26 | (-0.85, 0.34) | 0.115 | -0.05 | (-0.40, 0.30) | 0.073 | -0.39 | (-0.76, 0.01) | 0.185 |
|  | Yes | 1.64 | (-0.79, 4.07) |  | 1.11 | (-0.20, 2.42) |  | 0.43 | (-0.81, 1.66) |  |
| **Housing** | |  |  |  |  |  |  |  |  |  |
| Housing problems (number) | |  |  |  |  |  |  |  |  |  |
|  | 0 | -0.43 | (-1.08, 0.22) | 0.075 | -0.15 | (-0.54, 0.25) | 0.253 | -0.43 | (-0.82, -0.04) | 0.184 |
|  | 1 | -0.13 | (-0.89, 0.63) |  | 0.08 | (-0.38, 0.54) |  | -0.37 | (-0.84, 0.11) |  |
|  | 2 | 0.65 | (-0.32, 1.63) |  | 0.25 | (-0.31, 0.81) |  | 0.13 | (-0.51, 0.78) |  |
|  | 3+ | 1.07 | (-0.64, 2.78) |  | 0.66 | (-0.35, 1.68) |  | 0.26 | (-0.67, 1.19) |  |
| Easy internet access | |  |  |  |  |  |  |  |  |  |
|  | No | 0.72 | (-3.73, 5.19) | 0.723 | 1.10 | (-1.39, 3.59) | 0.389 | -0.51 | (-3.10, 2.08) | 0.847 |
|  | Yes | -0.07 | (-0.66, 0.51) |  | 0.02 | (-0.33, 0.36) |  | -0.26 | (-0.62, 0.10) |  |
|  | |  |  |  |  |  |  |  |  |  |
| **Relationships** | |  |  |  |  |  |  |  |  |  |
| Change in family relationships | |  |  |  |  |  |  |  |  |  |
|  | Lot worse | 5.39 | (1.10, 9.69) | **0.011** | 3.55 | (1.22, 5.89) | **0.007** | 1.83 | (-0.37, 4.04) | 0.075 |
|  | Little worse | 0.66 | (-0.35, 1.66) |  | 0.31 | (-0.30, 0.92) |  | 0.13 | (-0.54, 0.80) |  |
|  | Same | -0.40 | (-1.04, 0.23) |  | -0.18 | (-0.56, 0.20) |  | -0.38 | (-0.77, 0.01) |  |
|  | Little better | 0.04 | (-0.73, 0.82) |  | 0.13 | (-0.35, 0.61) |  | -0.24 | (-0.72, 0.24) |  |
|  | Lot better | -1.29 | (-2.82, 0.25) |  | -0.59 | (-1.44, -0.25) |  | -0.95 | (-1.81, -0.10) |  |
| Freq. argue with parents | |  |  |  |  |  |  |  |  |  |
|  | Never | -0.29 | (-1.52, 0.93) | **0.001** | -0.09 | (-0.70, 0.65) | 0.222 | -0.41 | (-1.08, 0.47) | **0.001** |
|  | Hardly ever | -1.03 | (-1.73, -0.32) |  | -0.32 | (-0.75, 0.12) |  | -0.88 | (-1.33, -0.42) |  |
|  | < once a week | 0.63 | (-0.35, 1.61) |  | 0.30 | (-0.27, 0.87) |  | 0.15 | (-0.40, 0.70) |  |
|  | > once a week | 0.16 | (-0.64, 0.96) |  | 0.11 | (-0.40, 0.61) |  | -0.09 | (-0.59, 0.43) |  |
|  | Most days | 0.62 | (-0.41, 1.64) |  | 0.16 | (-0.38, 0.69) |  | 0.21 | (-0.61, 1.04) |  |
| Feel Lonely | |  |  |  |  |  |  |  |  |  |
|  | Not at all | -1.28 | (-1.98, -0.58) | **<0.001** | -0.60 | (-1.01, -0.18) | **<0.001** | -0.84 | (-1.28, -0.41) | **<0.001** |
|  | Slightly | 0.17 | (-0.47, 0.81) |  | 0.12 | (-0.27, 0.50) |  | -0.12 | (-0.53, 0.29) |  |
|  | Moderately | 1.22 | (0.03, 2.40) |  | 0.64 | (-0.03, 1.32) |  | 0.40 | (-0.29, 1.10) |  |
|  | Very | 1.59 | (0.30, 2.88) |  | 1.25 | (0.50, 2.01) |  | 0.20 | (-0.67, 1.08) |  |
| **Routines** | |  |  |  |  |  |  |  |  |  |
| Stable routine | |  |  |  |  |  |  |  |  |  |
|  | Not at all | 1.11 | (0.05, 2.17) | **<0.001** | 0.80 | (0.21, 1.39) | **<0.001** | 0.14 | (-0.50, 0.78) | 0.149 |
|  | A little | 0.24 | (-0.60, 1.08) |  | 0.25 | (-0.22, 0.71) |  | -0.17 | (-0.68, 0.35) |  |
|  | A moderate amount | -0.38 | (-1.13, 0.36) |  | -0.16 | (-0.64, 0.31) |  | -0.44 | (-0.90, 0.03) |  |
|  | A lot | -1.74 | (-2.73, -0.78) |  | -0.99 | (-1.55, -0.42) |  | -0.86 | (-1.57, -0.15) |  |
|  | A great deal | -1.29 | (-2.45, -0.10) |  | -0.77 | (-1.45, -0.08) |  | -0.61 | (-1.30, 0.08) |  |
| Difficulty sleeping | |  |  |  |  |  |  |  |  |  |
|  | Not at all | -1.08 | (-1.74, -0.43) | **<0.001** | -0.44 | (-0.83, -0.04) | **0.002** | -0.80 | (-1.21, -0.39) | **0.004** |
|  | Yes a little | -0.01 | (-0.89, 0.88) |  | -0.03 | (-0.55, 0.48) |  | -0.13 | (-0.65, 0.40) |  |
|  | Yes sometimes | 0.61 | (-0.46, 1.67) |  | 0.45 | (-0.17, 1.07) |  | -0.01 | (-0.63, 0.61) |  |
|  | Yes quite often | 0.38 | (-0.51, 1.27) |  | 0.16 | (-0.40, 0.73) |  | 0.06 | (-0.54, 0.66) |  |
|  | Yes a lot | 1.56 | (0.21, 2.92) |  | 0.95 | (0.13, 1.78) |  | 0.37 | (-0.50, 1.24) |  |
| Change in exercise | |  |  |  |  |  |  |  |  |  |
|  | Much less | 1.34 | (0.44, 2.24) | **<0.001** | 0.82 | (0.27, 1.38) | **<0.001** | 0.31 | (-0.23, 0.84) | **0.036** |
|  | A bit less | 0.78 | (-0.30, 1.87) |  | 0.50 | (-0.10, 1.10) |  | 0.15 | (-0.49, 0.79) |  |
|  | No change | -0.76 | (-1.60, 0.08) |  | -0.16 | (-0.65, 0.32) |  | -0.72 | (-1.23, -0.21) |  |
|  | A bit more | -0.88 | (-1.66, -0.11) |  | -0.46 | (-0.93, 0.01) |  | -0.62 | (-1.10, -0.13) |  |
|  | Much more | -0.38 | (-1.26, 0.50) |  | -0.29 | (-0.87, 0.30) |  | -0.26 | (-0.83, 0.31) |  |
| **Summary variables** | |  |  |  |  |  |  |  |  |  |
| Index of negative impacts | |  |  |  |  |  |  |  |  |  |
| 0 | | -0.69 | (-1.35, -0.02) | **<0.001** | -0.25 | (-0.65, 0.15) | **<0.001** | -0.55 | (-0.98, -0.13) | **<0.001** |
| 1 | | -0.17 | (-0.93, 0.60) |  | -0.10 | (-0.55, 0.35) |  | -0.27 | (-0.72, 0.17) |  |
| 2 | | 0.07 | (-1.09, 1.22) |  | 0.12 | (-0.57, 0.80) |  | -0.19 | (-0.84, 0.47) |  |
| 3 | | 0.75 | (-0.87, 2.36) |  | 0.28 | (-0.68, 1.25) |  | 0.29 | (-0.88, 1.46) |  |
| 4 | | 1.08 | (-1.37, 3.54) |  | 0.84 | (-0.58, 2.27) |  | -0.02 | (-1.51, 1.47) |  |
| 5 | | 0.70 | (-1.92, 3.33) |  | 0.49 | (-0.83, 1.80) |  | -0.06 | (-1.95, 1.83) |  |
| 6 | | 2.42 | (0.24, 4.60) |  | 2.02 | (0.49, 3.56) |  | 0.31 | (-0.96, 1.58) |  |
|  | |  |  |  |  |  |  |  |  |  |
| Index of negative impacts (count) | | 0.43* | (0.22, 0.65) | **<0.001** | 0.24 | (0.11, 0.36) | **<0.001** | 0.19 | (0.04, 0.33) | **0.011** |
| Number of substantive concerns | | 0.16* | (0.08, 0.24) | **<0.001** | 0.10 | (0.05, 0.14) | **<0.001** | 0.06 | (0.01, 0.11) | **0.013** |
| Number of positives | | -0.17* | (-0.28, -0.06) | **0.002** | -0.09 | (-0.15, -0.02) | **0.008** | -0.08 | (-0.15, -0.02) | **0.010** |

All estimates are weighted, using inverse probability weights, and robust standard errors are used throughout. Model adjusted for age and days passed since data collection (entered as a linear and quadratic term). b, regression coefficient. * Coefficients are interaction terms and indicate the amount by which change in SDQ from pre- to post-Covid increases or decreases for each additional impact, concern, or positive.
